# *Arabidopsis thaliana* population analysis reveals high plasticity of the genomic region spanning *MSH2, AT3G18530* and *AT3G18535* genes and provides evidence for NAHR-driven recurrent CNV events occurring in this location

**DOI:** 10.1186/s12864-016-3221-1

**Published:** 2016-11-08

**Authors:** Agnieszka Zmienko, Anna Samelak-Czajka, Piotr Kozlowski, Maja Szymanska, Marek Figlerowicz

**Affiliations:** 1 Institute of Bioorganic Chemistry, Polish Academy of Sciences, Noskowskiego 12/14, 61-704 Poznan, Poland; 2 Institute of Computing Science, Poznan University of Technology, Piotrowo 2, 60-965 Poznan, Poland

**Keywords:** Copy number variation (CNV), Multiallelic CNV, Non-allelic homologous recombination (NAHR), Recurrent deletion, Multiplex ligation-dependent probe amplification (MLPA), Droplet digital PCR, Genotyping, *Arabidopsis thaliana*

## Abstract

**Background:**

Intraspecies copy number variations (CNVs), defined as unbalanced structural variations of specific genomic loci, ≥1 kb in size, are present in the genomes of animals and plants. A growing number of examples indicate that CNVs may have functional significance and contribute to phenotypic diversity. In the model plant *Arabidopsis thaliana* at least several hundred protein-coding genes might display CNV; however, locus-specific genotyping studies in this plant have not been conducted.

**Results:**

We analyzed the natural CNVs in the region overlapping *MSH2* gene that encodes the DNA mismatch repair protein, and *AT3G18530* and *AT3G18535* genes that encode poorly characterized proteins. By applying multiplex ligation-dependent probe amplification and droplet digital PCR we genotyped those genes in 189 *A. thaliana* accessions. We found that *AT3G18530* and *AT3G18535* were duplicated (2–14 times) in 20 and deleted in 101 accessions. *MSH2* was duplicated in 12 accessions (up to 12-14 copies) but never deleted. In all but one case, the *MSH2* duplications were associated with those of *AT3G18530* and *AT3G18535*. Considering the structure of the CNVs, we distinguished 5 genotypes for this region, determined their frequency and geographical distribution. We defined the CNV breakpoints in 35 accessions with *AT3G18530* and *AT3G18535* deletions and tandem duplications and showed that they were reciprocal events, resulting from non-allelic homologous recombination between 99 %-identical sequences flanking these genes. The widespread geographical distribution of the deletions supported by the SNP and linkage disequilibrium analyses of the genomic sequence confirmed the recurrent nature of this CNV.

**Conclusions:**

We characterized in detail for the first time the complex multiallelic CNV in Arabidopsis genome. The region encoding *MSH2*, *AT3G18530* and *AT3G18535* genes shows enormous variation of copy numbers among natural ecotypes, being a remarkable example of high Arabidopsis genome plasticity. We provided the molecular insight into the mechanism underlying the recurrent nature of *AT3G18530*-*AT3G18535* duplications/deletions. We also performed the first direct comparison of the two leading experimental methods, suitable for assessing the DNA copy number status. Our comprehensive case study provides foundation information for further analyses of CNV evolution in Arabidopsis and other plants, and their possible use in plant breeding.

**Electronic supplementary material:**

The online version of this article (doi:10.1186/s12864-016-3221-1) contains supplementary material, which is available to authorized users.

## Background

The rapid improvement of high throughput sequencing methods and the consecutive boost in genomic studies over the past few years have revealed the unexpectedly wide extent of intraspecies structural variation in the genomes of both animals and plants. Copy number variation (CNV) is a type of genetic polymorphism manifested through varying copy numbers of large genome fragments (typically more than 1 kb) [1]. CNV regions often span protein coding genes [2–7]. Changes in the number of functional gene copies (or their distal regulatory regions) might affect the amount of expressed protein and consequently alter the phenotype. Indeed, recent reports have clearly shown that in humans, CNV substantially impacts genome evolution, phenotypic variation and adaptation [8–11]. Similarly, the importance of CNV for plant fitness has been vividly demonstrated, e.g., the rapid (within a decade) spreading of glyphosate resistance among American populations of the weed plant, palmer amaranth (*Amaranthus palmeri*), resulting from the amplification of the gene encoding 5-enolpyruvylshikimate 3-phosphate (EPSP) synthase, which is targeted by this herbicide [12]. Additionally, increased resistance to soybean cyst nematode (SCN) reported in some soybean (*Glycine max*) lines has been associated with the duplication of the genomic region *Rhg1*, which spans 3 genes likely involved in counteracting the pathogen infection [13]. Some maize (*Zea mays*) lines have superior tolerance to high aluminum ion concentration, reflecting the triplication of the *MATE-1* gene, which encodes an anion transporter, while in barley (*Hordeum vulgare*), the amplification of the boron transporter gene *Bot1* increased plant tolerance to boron toxicity [14, 15]. Other examples of plant CNVs also concern crops and traits important for plant breeding [16]. CNV has been associated with differences in flowering time in several species, including wheat (*Triticum aestivum),* barley and rapeseed (*Brassica napus*) [17–21]. The rice (*Oryza sativa)* landrace Ping13 has a superior grain length and quality that reflects the tandem duplication of the *GL7* gene, which encodes a protein homologous to Arabidopsis (*Arabidopsis thaliana*) LONGIFOLIA proteins [22].

These observations highlight the need for the systematic discovery and validation of genes that undergo CNV to characterize the mechanisms driving CNV formation in plants. To date, the molecular studies regarding this issue are scarce. In soybean, phylogenetic analysis revealed the common origin of SCN resistance from a single progenitor, followed by subsequent copy number expansion (tandem duplications) and the divergence of the *Rhg1* locus [23]. The duplicated copies of the gene encoding EPSP synthase are spread throughout the genome of palmer amaranth; therefore, the involvement of transposable elements in CNV formation has been assumed [12]. In both cases, strong positive selection pressure contributed to the spread of gene duplications. Moreover, genetic studies have revealed multiple examples of recurrent CNVs present in human populations, primarily triggered through the genomic architecture. One mechanism shown to be involved in the formation of recurrent CNVs in humans is non-allelic homologous recombination (NAHR) occurring between regions (typically longer than 1 kb) of high sequence homology (over 95 %), known as segmental duplications or low copy repeats (LCRs) [24, 25]. Considering the numerous large-scale genome events, such as polyploidy, or duplications of large chromosomal regions in plants [26–28], NAHR might also be expected to significantly contribute to CNV formation in plants.

Based on preliminary data from the Arabidopsis 1001 Genomes Project, the draft map of CNVs in this model plant was generated [29]. This effort has been the only population-scale genome-wide CNV detection study in this plant published to date. The map was based on whole-genome sequencing (WGS) data of 80 natural accessions, originating from various geographic locations in Europe, Asia and North Africa (MPICao2010 set). Using the read depth analysis, the authors inferred 1,059 CNVs (1 – 13 kb long), covering 1.8 % of the Arabidopsis reference genome and partially or completely spanning more than 500 protein-encoding genes. From this dataset, we selected a region, encompassing 3 genes (*MSH2/AT3G18524*, *AT3G18530* and *AT3G18535*), where Cao et al. inferred high-level duplications and frequent deletions [29]. *MSH2* codes for an Arabidopsis homolog of the MutS protein, an essential component of the DNA mismatch repair system [30, 31], while *AT3G18530* and *AT3G18535* encode proteins with poor functional annotation. We analyzed the sequence of this genomic region in the reference genome (TAIR10) and observed that *AT3G18530* and *AT3G18535* are flanked by 1 kb long direct repeats of 99 % identity which suggested the possible involvement of the NAHR mechanism in CNV formation. In contrary to human genome where numerous NAHR-derived CNVs are under deep investigation [25, 32–34], we are not aware of similar studies performed in plants. Due to the high level of variation suggested by the WGS data [29] the region encompassing *AT3G18530* and *AT3G18535* genes could be therefore used as a great model to study NAHR in plants and to examine the influence of selection pressure on the evolution of a highly recombinogenic region. As a first step to evaluate this possibility, in the present study we calculated the copy numbers of *MSH2, AT3G18530* and *AT3G18535* genes in 189 Arabidopsis accessions. We analyzed the chromosome breakpoints in accessions with the *AT3G18530* and *AT3G18535* tandem duplications/deletions and we confirmed the involvement of the NAHR mechanism in the origin of these variations. By evaluating the geographical distribution of the identified genotypes, supported by the SNP analysis we revealed the recurrent nature of the analyzed CNV. Moreover, we directly compared the accuracy of two popular methods of locus-specific CNV analysis: multiplex ligation-dependent probe amplification (MLPA) [35] and droplet digital PCR (ddPCR) [36–38], which we used for the first time for CNV genotyping in plants. The present study is the first locus-specific analysis of a CNV region in Arabidopsis, providing insight into the mechanisms and factors driving CNV evolution and describing the successful adaptation of popular genotyping tools for plant genome research.

## Results

### *MSH2*, *AT3G18530* and *AT3G18535* genes undergo CNV in Arabidopsis population

**Fig. 1:**
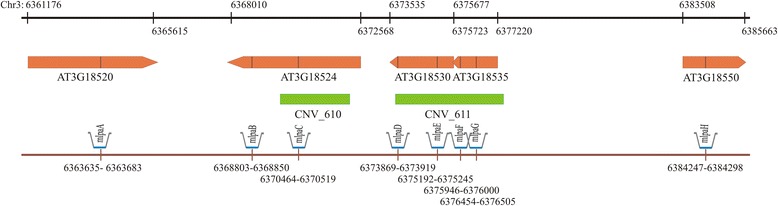
The structure of Arabidopsis genomic region covered by the Ath_CNV610-611_MLPA assay. On the black axis the coordinates of the target genes (marked in orange below the axis) are given. On the brown axis the localization of each MLPA probe (mlpaA-mlpaH) is indicated. Probes mlpaC-mlpaG are located within CNV regions (marked in green). The size and distance between the elements in the figure are scaled

According to Cao et al. [29], the *AT3G18530* and *AT3G18535* genes and a part of the *MSH2* gene are covered by two distinct CNVs (CNV_611 and CNV_610) separated by a 1.5-kb distance (Fig. 1). The two CNVs differed according to the frequency of copy number alterations (multiple instances of duplication/deletion at CNV_611 and only 4 accessions with duplications at CNV_610). In addition, we observed some interesting interrelatedness – the duplication at CNV_610 was accompanied by similar levels of duplication at CNV_611 (Additional file 1: Figure S1). We analyzed the DNA copy number in this region, focusing on the protein-coding genes. The analysis comprised a set of 189 Arabidopsis accessions from the Arabidopsis 1001 Genomes Project [39]. Our experimental set included all 80 accessions represented in the MPICao2010 set and 109 accessions from the Salk collection. As a control and a calibrator with 2 copies per diploid genome of each analyzed gene, we used Col-0, which also served as a reference accession in the WGS-based CNV genotyping study [29].

MLPA is one of the most widely used methods of CNV genotyping in humans. In MLPA, each individual probe is divided into two fragments (half-probes), which are ligated and amplified only if these fragments hybridize to the analyzed region. The amount of the amplified product, determined through capillary electrophoresis, reflects the dosage of the DNA template. MLPA assays can be multiplexed after designing probes that generate products of a unique length, visible as separate peaks on electropherograms. We have previously optimized an approach based on synthetic oligonucleotides for the easy design of MLPA probes for genotyping numerous human CNVs [40, 41]. Here, we applied this method to design the Ath_CNV610-611_MLPA probe set to target the genes of interest (Fig. 1 and Additional file 1: Figure S2). Two MLPA probes were designed for each gene: mlpaB and mlpaC for *MSH2*, mlpaD and mlpaE for *AT3G18530,* and mlpaF and mlpaG for *AT3G18535*. For *MSH2*, one probe from the pair was located within CNV_610 (which overlaps exons 3–6), while the second probe was located in exon 11, outside of the predicted CNV. Additional probes were designed for genes located outside the inferred CNVs, flanking the variable DNA segment: mlpaA for *HDA15* (*AT3G18520*) and mlpaH for *BRC1* (*AT3G18550*). Altogether, the Ath_CNV610-611_MLPA assay covered 25.5 kb of the Arabidopsis genome. We also designed 5 probes, each targeting one protein-coding gene with stable copy numbers (according to WGS analysis) located on different chromosomes. The respective probes (ctrl1-ctrl5) were generated to cover the entire range of DNA fragment lengths amplified in the Ath_CNV610-611_MLPA multiplex assay (96–172 bp) and served as internal controls (Table 1).

**Table 1:** Probes used in the Ath_CNV610-611_MLPA assay and their target genes

Probe	Locus	Gene product	Amplified fragment length (bp)
mlpaA	AT3G18520	HDA15, protein with similarity to histone deacetylases	108
mlpaB	AT3G18524	MSH2, MutS protein homolog 2, involved in maintaining genome stability and repressing recombination of mismatched heteroduplexes	152
mlpaC			128
mlpaD	AT3G18530	ARM repeat superfamily protein	168
mlpaE			117
mlpaF	AT3G18535	Tubulin-tyrosine ligase	160
mlpaG			99
mlpaH	AT3G18550	BRC1, a TCP transcription factor, arrests axillary bud development and prevents axillary bud outgrowth	136
ctrl1	AT1G01040	DCL1, a RNA helicase involved in microRNA processing	96
ctrl2	AT4G21580	oxidoreductase, a zinc-binding dehydrogenase family protein	111
ctrl3	AT2G36230	APG10, a BBMII isomerase involved in histidine biosynthesis	124
ctrl4	AT5G23290	PFD5, a prefoldin involved in unfolded protein binding	144
ctrl5	AT1G73010	PS2, a pyrophosphate-specific phosphatase	172

**Fig. 2:**
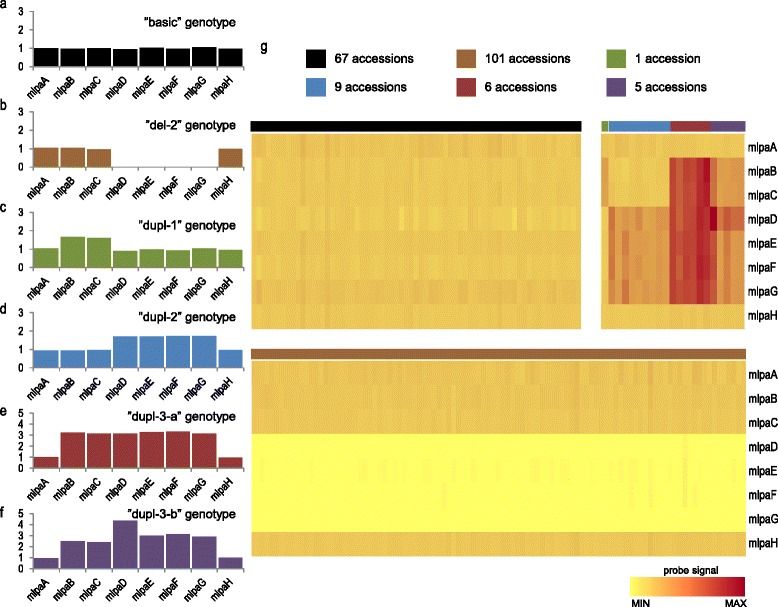
CNV patterns detected by the Ath_CNV610-611_MLPA assay. a-f, probe signal plots representative of accessions with: no copy number changes - “basic” genotype (**a**), deletion of *AT3G18530* and *AT3G18535* – “del-2” genotype (**b**), duplication of *MSH2* – “dupl-1” genotype (**c**), duplication of *AT3G18530* and *AT3G18535* - “dupl-2” genotype (**d**), duplication of *MSH2*, *AT3G18530* and *AT3G18535* with equal signals from all probes - “dupl-3-a” genotype (**e**) or duplication of *MSH2*, *AT3G18530* and *AT3G18535* with increased signal from probe mlpaD - “dupl-3-b” (**f**). g, heatmaps of probe signals (rows) in accessions (columns) grouped by the CNV pattern. Color bars above the heatmaps indicate the genotypes represented by plots (**a**-**f**). Data on plots (a-f) are from accessions: Lag2-2, Vie-0, Kl-5, La-0, Uod-1 and Bak-7 respectively. See Table 1 for the description of MLPA probes

Each MLPA probe in this assay produced a well-resolved peak at the expected position (migration rate) in the electropherograms (Additional file 1: Figure S3). Changes in the gene copy number within the analyzed set of accessions were detected through the comparison of the normalized peak heights for each probe (Fig. 2). As expected, no CNV was observed for the *HDA15* and *BRC1* genes and the regions selected as controls in any accession. In 67 accessions, the copy numbers for *MSH2*, *AT3G18530* and *AT3G18535* genes were similar to those detected in Col-0; therefore, we referred to this genotype as “basic”. In these samples, all probes (mlpaA-mlpaH, ctrl1-ctrl5) presented low signal variations (SD < 10 %), indicative of reliable and consistent performance [42]. As many as 101 accessions (53.4 %) harbored deletions of *AT3G18530* and *AT3G18535* (genotype “del-2”). Duplications of at least one gene were detected in the remaining accessions, and several duplication patterns were observed. In 1 accession, *MSH2* was the only duplicated gene (genotype “dupl-1”). In 9 accessions, *AT3G18530* and *AT3G18535* genes were duplicated (genotype “dupl-2”)*.* In 6 accessions, *MSH2, AT3G18530* and *AT3G18535* genes were duplicated (genotype “dupl-3-a”). In the remaining 5 accessions, the duplication of all 3 genes was also observed, but the signal from probe mlpaD was markedly higher than the signals from other probes (genotype “dupl-3-b”). Regardless of the latter, the signal correlations of MLPA probe pairs specific for particular genes were high, as were the pairwise signal correlations of all 4 probes covering the block of *AT3G18530* and *AT3G18535* genes (Additional file 1: Figure S4). A higher divergence of the outermost probes mlpaD and mlpaG might reflect the variations observed in the sequence and/or location of CNV breakpoints among the individual DNA copies, thereby affecting MLPA probe hybridization. Thus, we concluded that *AT3G18530* and *AT3G18535* overlap a single CNV, and we inferred the same copy number genotypes for both genes in all analyzed accessions using mlpaE and mlpaF probes (see below).

### Copy numbers of *MSH2*, *AT3G18530* and *AT3G18535* genes in Arabidopsis population

**Fig. 3:**
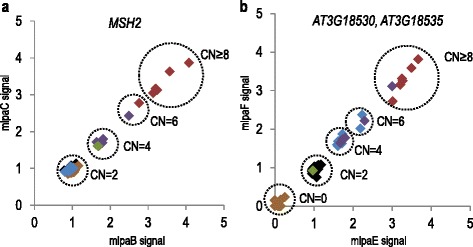
Clusters of Arabidopsis accessions containing different gene copy numbers identified with Ath_CNV610-611_MLPA assay. The scatterplots present signals of paired MLPA probes for: **a** *MSH2* and **b** *AT3G18530*-*AT3G18535*. All results were calibrated using data obtained for Col-0 accession. The clustering and copy number (CN) assignment was done manually. The data points (accessions) are colored according to the CNV patterns described in Fig. 2

In previous reports, we showed that the copy numbers of the individual genes could be accurately determined after analyzing the data obtained for two independent MLPA probes on two-dimensional plots [41, 43]. Such plots were generated for the probes targeting *MSH2* (mlpaB-mlpaC) and the *AT3G18530*-*AT3G18535* block (mlpaE-mlpaF). The results showed that clusters of accessions with different copy numbers could be easily distinguished (Fig. 3). As Arabidopsis is primarily self-pollinating and laboratory-maintained plants are typically homozygous, we assumed that subsequent clusters corresponded to genotypes differing by two copies. For *MSH2,* we distinguished 4 clusters with the following copy numbers: 2 gene copies (in 177 accessions), 4 copies (in 5 accessions), 6 copies (in 2 accessions) and at least 8 copies (in 5 accessions). For *AT3G18530* and *AT3G18535*, we observed 5 clusters. The apparent lack of a signal from both probes indicated gene deletion in 101 accessions. In 68 accessions, 2 copies were detected. In the remaining cases, duplications were observed: 4 copies in 10 accessions, 6 copies in 3 accessions and at least 8 copies in 7 accessions.

**Fig. 4:**
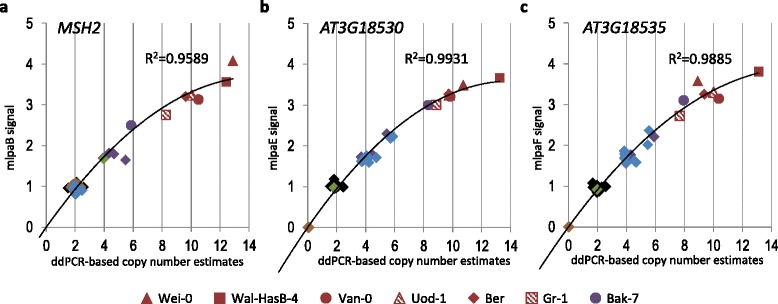
Concordance of MLPA-based and ddPCR-based gene copy number genotyping results in 92 accessions. On x-asis, ddPCR-based absolute gene copy numbers are shown. On y-axis, normalized MLPA signals are shown, generated with the MLPA probe located nearest to the ddPCR primers’ target position (Additional file 1: Figure S2). The data points (accessions) are colored according to the CNV patterns described in Fig. 2 and accessions with the highest levels of duplication are given unique symbols. Weaker data correlation observed for *MSH2* in comparison with *AT3G18530* and *AT3G18535* is caused by the low number of accessions (12 out of 92 presented) with *MSH2* gene copy number other than 2

For MLPA assays, the distance between the clusters decreases with increasing integer copy number [41]. Therefore, we did not attempt to partition the relatively small clusters of accessions containing 8 or more copies of *MSH2* and *AT3G18530*-*AT3G18535* genes. To calculate the number of gene copies in the accessions with the highest level of duplications, we applied ddPCR. In this approach, the DNA template is highly diluted, facilitating the amplification of single copies of the target region, distributed among thousands of independent reaction partitions (droplets). After applying Poisson statistics, the copies of targeted DNA are quantified after counting the positive (amplification signal detected) and negative (no signal) reaction droplets. In the present study, we used gene-specific primers to analyze all 21 accessions with “dupl-1”, “dupl-2”, “dupl-3-a” and “dupl-3-b” genotypes, 48 randomly selected accessions with the “del-2” genotype and 23 randomly selected accessions with the “basic” genotype. The calculated gene copy numbers were compared with the MLPA data. Both methods were entirely consistent in the identification of copy number variation (duplication/deletion/no change). The precise ddPCR-calculated copy numbers and MLPA probe intensities were highly but nonlinearly correlated, clearly revealing that ddPCR has much better resolution for genotyping highly duplicated genes (Fig. 4). Accordingly, ddPCR facilitates a more accurate determination of the gene copy numbers in the 7 accessions that could not be precisely characterized with MLPA. The estimated gene copy number in those accessions ranged from 8 to 12–14 copies.

### Geographic distribution of CNV genotypes

**Fig. 5:**
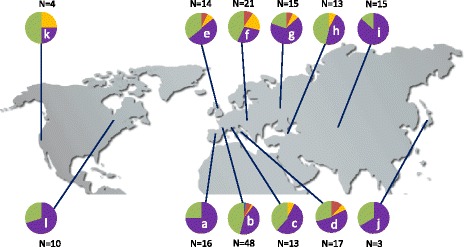
Geographic distribution of the CNVs for the genomic region spanning *MSH2*, *AT3G18530* and *AT3G18535* genes. The number of accessions sampled for each region (N) is reported. Colors indicate genotypes: “basic” (green); “del-2” (purple), “dupl-1” (blue), “dupl-2” (red), “dupl-3-a” or “dupl-3-b” (yellow). a - Iberian Peninsula & Morocco, b - Western Europe, c – Alps, d – Italy, e - Northern Europe, f - Central & Southeast Europe, g - Eastern Europe, h - Western Asia & Caucasus, i - Central Asia, j - East Asia, k - Pacific Northwest, l – Midwest

**Fig. 6:**
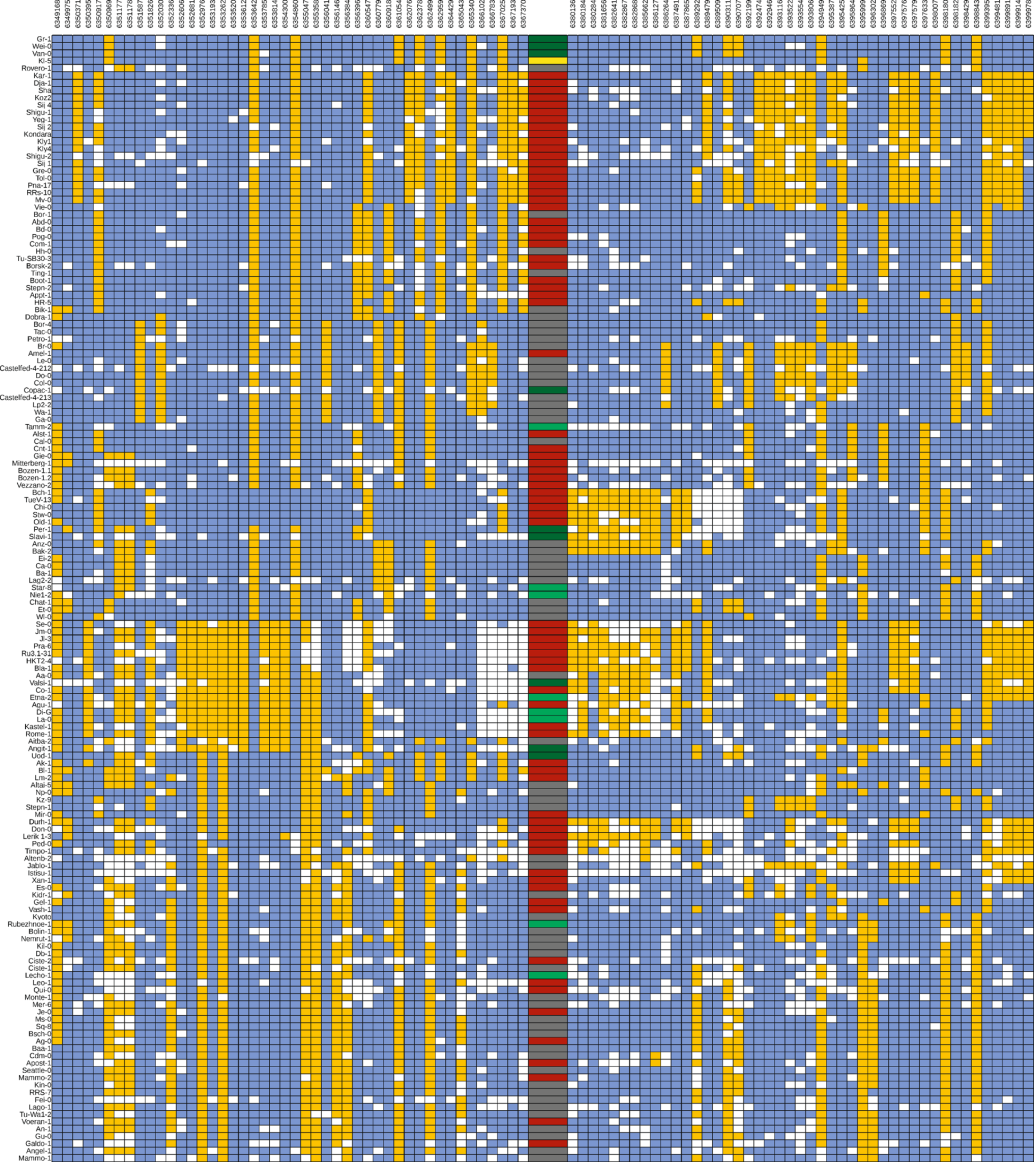
Haplotypes determined for the genomic regions surrounding *MSH2*, *AT3G18530* and *AT3G18535* loci, for 154 accessions. Bi-allelic SNPs of at least 10 % frequency located in 20-kb regions from both sides of the investigated CNV were analyzed. Dominant genotype in each position is marked in blue, alternative genotype is marked in yellow. SNP genomic coordinates are indicated on the top. CNV genotypes are marked as dark grey (“basic”), red (“del-2”) yellow (“dupl-1”), green (“dupl-2”) and dark green (“dupl-3-a” and “dupl-3-b”, collectively). The order of accessions reflects the distance-based tree generated in SplitsTree program

The samples analyzed in the present study originated from various locations: 143 accessions from Europe, 31 accessions from Asia, 14 accessions from North America and 1 accession from North Africa (Morocco) (Additional file 2: Table S1). The most frequent genotype, “del-2”, was detected in accessions from all investigated geographic regions (Fig. 5). In contrast, “dupl-1”, “dupl-2”, “dupl-3-a” and “dupl-3-b” patterns were primarily spread across various parts of Europe. Single accessions with the “dupl-3-a” and “dupl-3-b” genotypes were also detected in North America and Western Asia, respectively. Non-European accessions constituted only 23.8 % of the experimental set, and the duplications were associated with the least abundant genotypes, even across Europe. We therefore consider it highly likely that sampling additional non-European accessions would reveal additional examples of “dupl-1” and “dupl-2” genotypes within these groups. Although we did not observe any correlation between the genotype and geographic origin of the plants, to ascertain whether plants with particular CNV variants might originate from common ancestors, we analyzed the bi-allelic SNPs of at least 10 % frequency in the 20-kb genomic regions flanking the investigated segment from both sides in a subset of 153 accessions for which well-validated SNP data has been released recently by 1001 Genomes Consortium [44] and additionally in Col-0. We constructed the phylogenetic network using the distance-based method NeighborNet, implemented in SplitsTree [45]. We then compared the distribution of the CNV genotypes with the genetic groups identified by whole-genome variation analysis of 1,135 Arabidopsis accessions [44]. The analysis did not reveal any clear evolutionary splits between accessions harboring distinct CNV genotypes (Additional file 1: Figure S5). One remarkably distinguishing network branch included accessions from Kyrgyzstan, Russia, Tajikistan, Uzbekistan, Armenia and USA, which all harbored “del-2” genotype. However, these Eurasian accessions were previously shown to present the lowest level of allele differentiation and the smallest number of private SNPs [29, 46]. Also, according to recent phylogenetic data, the USA accessions belong to Germany genetic cluster and exhibit extensive haplotype sharing [44]. We did not detect the relationship between the CNV patterns and any other haplotypes (Fig. 6). We further analyzed the linkage disequilibrium (LD) of bi-allelic SNPs and CNV genotypes. Two regions of high LD were detected on both sides of the CNV segment, but similarly, no correlation between any SNP and any CNV pattern was observed (R^2^ < 0.3) (Additional file 1: Figure S6). Altogether these observations supported the hypothesis that *MSH2* and/or *AT3G18530-AT3G18535* copy number changes occurred independently in multiple accessions.

### Prevalence of “del-2” genotype in Arabidopsis population

The depth-of-coverage methods rely on counting the number of reads mapping to a particular genomic position to ascertain the DNA copy number and detect CNV regions. We analyzed the genomic sequence of *AT3G18530-AT3G18535* loci in the recently released pseudogenome sequences for 1135 Arabidopsis accessions [44]. These pseudogenomes were generated based on WGS data, by combining reference and variant calls including indels, with uncalled sites represented as Ns. We observed that 687 accessions had at least 80 % values missing in the analyzed region (Additional file 1: Figure S7). These included all accessions with “del-2” genotype, for which pseudogenomes were available (76 cases). On the contrary, none of the 18 accessions with the duplication genotypes and available pseudogenomes was found in this group. Likewise, only 2 out of 59 accessions with “basic” genotype had at least 80 % missing values at this region. Thus, we concluded that the presence of the “del-2” genotype could be predicted with high efficiency and low false positive error from the missing values in the WGS data. Consequently, we estimated that the frequency of “del-2” genotype among 1,135 Arabidopsis accessions was 60.53 %. We further determined the “del-2” frequency in 10 countries with the highest sample representation in the above collection (Table 2). It ranged from 46.6 % in Germany to 80.56 % in Spain. Thus, we propose that the CNV of this genomic segment is widespread throughout the Arabidopsis population; in particular, *AT3G18530* and *AT3G18535* are frequently deleted.

**Table 2:** Comparison of the frequency of “del-2” genotype across the countries, inferred from the analysis of Arabidopsis 1,001 Genomes Project WGS data and experimentally determined in current study

Country	Number of representing accessions		Frequency of “del-2” genotype	
	1,001 Genomes Project	This study	1,001 Genomes Project^a^	This study
All countries	1135	189	60.61 %	53.44 %
Highly represented countries (listed below):	979	128	64.04 %	56.25 %
Sweden	243	1	55.97 %	0.00 %
Spain	180	10	80.56 %	100.00 %
USA	123	12	86.99 %	58.33 %
Germany	118	32	46.61 %	37.50 %
Italy	73	28	47.95 %	53.57 %
United Kingdom	69	10	71.01 %	60.00 %
Russia	60	16	80.00 %	75.00 %
France	45	10	46.67 %	70.00 %
Czech Republic	40	7	55.00 %	42.86 %
Bulgaria	28	2	32.14 %	0.00 %

^a^The “del-2” genotype was assigned to accessions with at least 80 % missing values in the sequence of the genomic region spanning *AT3G18530* and *AT3G18535* loci

### Sequence analysis of breakpoints in accessions with “del-2” and “dupl-2” genotypes

**Fig. 7:**
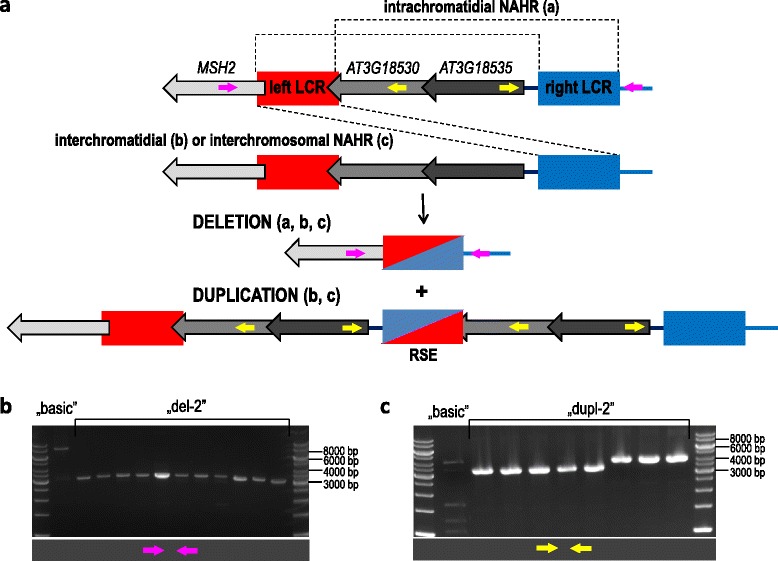
LCRs bordering *AT3G18530* and *AT3G18535* genes and their role in mediating NAHR-based gene copy number changes. a - Hypothesized model of nonhomologous pairing between the left (Chr3:6372413..6373650, red) and right (Chr3:6377368..6378605, blue) LCR, leading to deletion (in case of intrachromatidial NAHR) or to deletion and reciprocal duplication (in case of interchromatidial/interchromosomal NAHR) of the flanked genomic DNA. The actual site of strand exchange and the length of the conversion track determine the sequence of the repeat reconstituted after recombination: identical to one of the original LCRs or chimeric. Pink arrows show the estimate localizations of primers used for amplification of the breakpoint region in accessions with “del-2” genotype. Yellow arrows show the estimate localization of primers used for amplification of the breakpoint region in accessions with “dupl-2” genotype. RSE – region of strand exchange; b - Representative gel image of genomic DNA fragments amplified in accessions with “del-2” genotype and in Col-0 (“basic” genotype). The expected length of DNA amplicon is 3,421 bp (8,376 bp in case of Col-0); c - Gel image of genomic DNA fragments amplified in accessions with “dupl-2” genotype and in Col-0 (“basic” genotype). The expected length of DNA amplicon is 3,404 bp (no product in case of Col-0)

Our data indicated that *AT3G18530* and *AT3G18535* genes are located within a single CNV, and the individual genotypes are spread across various regions of the Arabidopsis habitat. Considering the high frequency of the “del-2” genotype in the global population, we concluded that the CNV spanning *AT3G18530* and *AT3G18535* genes might be recurrent. *MSH2*, *AT3G18530* and *AT3G18535* are all encoded on the minus strand of chromosome 3. In the reference genome, two 1238-bp LCRs of 99 % sequence identity flank the *AT3G18530*-*AT3G1853* gene block. The left LCR separates *MSH2* from *AT3G18530*, partially overlapping the first and last exon, of the former and latter gene, respectively. The right LCR is localized at 148 nt upstream of the *AT3G18535* gene start site (regarding the minus strand). Apart from those positions, the LCR sequence is not repeated elsewhere in the genome. We hypothesized that the natural variation of this region might be promoted through NAHR events, mediated through LCRs (Fig. 7a). According to the double strand break (DSB) repair model, intrachromatidal NAHR, involving a double Holliday junction (dHj), might lead to the deletion of *AT3G18530* and *AT3G18535* genes, while interchromosomal or interchromatidal NAHR generates deletions and reciprocal tandem duplications [24, 32]. Using DNA sequencing, we confirmed that both LCRs were present in the expected genomic positions in Col-0, and these sequences were identical to the reference genome. Subsequently, we amplified the deletion junction fragment in 27 accessions of various geographic origins harboring the “del-2” genotype. The lengths of the amplified products (approximately 3.4 kb in case of deletion versus approximately 8.4 kb in Col-0) were consistent with the above model, assuming the loss of a 5-kb region including both genes and one LCR (Fig. 7b). Similarly, we performed breakpoint-spanning PCR to detect the tail-to-head orientation of the *AT3G18535*-*AT3G18530* gene blocks with intervening LCR sequence in 8 lines with “dupl-2” genotypes. The presence of amplification products was confirmed for all accessions, although 3 of these products were longer (approximately 4.2 kb) than predicted (approximately 3.4 kb) (Fig. 7c). No amplification product was observed in the case of Col-0, as expected.

**Fig. 8:**
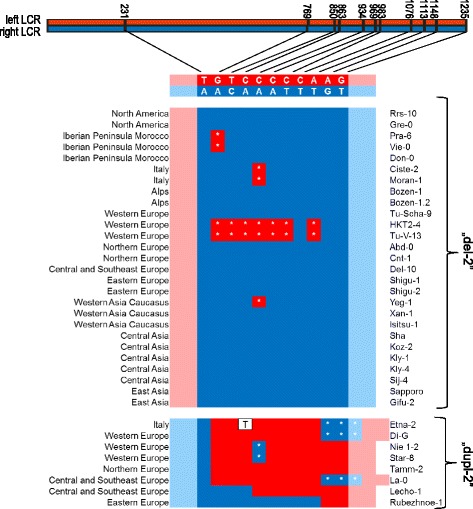
Sequence analysis of chromosome breakpoints in accessions with “del-2” and “dupl-2” genotypes. The 1238-bp long LCRs differ in 11 positions. Left and right LCR are colored in red and blue, respectively. The variable sites which differentiate both LCRs are indicated – they accumulate in the right half of the repeat. The upstream and downstream sequences directly adjacent to each LCR are marked in respective but less intensive color. White asterisks indicate gene conversion events. Single nucleotide substitution found in the variable site in one accession is presented on white background

The left and right LCRs differ by 11 nucleotides which divide this region of homology into 10 intervals and permit to map recombination breakpoints to short DNA stretches (Fig. 8). We partially sequenced the amplification products described above, confirming that the CNV breakpoint was indeed located within the LCR sequence. The switches between the sequences of the left and right LCRs in accessions with “del-2” genotype were localized between position 1 and 231 (the first interval), while the switches between the right and left LCR sequences in accessions with “dupl-2” genotype were mostly localized between position 231 and 769 (the second interval). The mutual arrangement between “del-2” and “dupl-2” genotypes accurately fits the NAHR model involving dHj, that is subsequently resolved by crossover (see [33] for the description and schematic representation of the dHj formation and resolution). Therefore it supports the reciprocity of the duplication/deletion events leading to both genotypes. According to the above model, the DSB site is located between the two sequence junction sites detected in accessions with “del-2” and “dupl-2” genotypes, respectively. In this case the DSB site could be tracked to the 5’ half of the LCR sequence (between position 1 and 769). This is the region of the longest uninterrupted homology between both LCRs (230 and 537 bp tracks, separated by only one mismatch, T/A). The track length is consistent with the range of minimal efficient processing segments (regions sharing extremely high similarity or identity necessary for NAHR to occur), as empirically estimated for human meiosis at 300 to 500 bp (reviewed in [24]). Additionally, in 7 accessions with the “del-2” genotype and in 5 accessions with the “dupl-2” genotype, the repeat sequence was a mixture of the left and right LCRs, reflecting discontinuous sequence conversion events [33]. In 3 accessions with duplications, the conversion track was extended approximately 790 bp beyond the right LCR, resulting in larger duplication. The presence and length of this extended duplication were consistent with the size of the amplification products detected on the gel electropherogram presented above. Interestingly, the sequence restoration in those accessions was likely mediated through the 9-nt microhomology between the duplicated fragment and the 3’ border of the left LCR (Additional file 1: Figures S8- S11). In addition, substantial sequence variation was observed around the breakpoint region, both in lines with “del-2” and with “dupl-2” genotype, consistent with previous findings for CNV in humans [47].

## Discussion

The information derived from high-throughput sequencing projects revealed that CNV affects a substantial part of plant genomes [48–50]. However, in plants, the CNV maps or detailed characteristics of individual CNVs are not abundant. Most notably, only one genome-wide CNV analysis has been performed in the top model plant Arabidopsis [29] and none of the identified loci have been studied in detail, apart from the brief verification of presence-absence variants [51]. Here, we presented the first detailed analysis of a complex CNV in the Arabidopsis genome with a wide range of changes in the copy number across individuals. This type of polymorphism is referred to as multiallelic CNV (mCNV). In humans, mCNVs have received much attention, reflecting the high prevalence of these changes in the genome and the fact that these variations frequently affect protein-coding genes. Examples of mCNVs that modify the human phenotype include the *CCL3L1* and *DEFB* genes, which are associated with infectious and inflammatory-related diseases [52]. Additionally, it was recently estimated that mCNVs contribute about 7 times more to the gene dosage variation than much more abundant simple biallelic CNVs [38].

The complex mCNV characterized in our study spans 3 protein-coding genes, *MSH2*, encoding a key component of the mismatch repair pathway, and *AT3G18530* and *AT3G18535*, for which little functional information exists. The experimental data obtained in the present study which involved 189 Arabidopsis accessions indicated that 53.4 % of them lost the DNA region harboring the latter genes. According to our analysis of WGS data available for 1,135 accessions form 1001 Genomes collection, this rate is even higher and over 60 % of accessions might actually harbor the “del-2” genotype. Therefore, *AT3G18530* and *AT3G18535* genes are not strictly essential for plant growth. The “del-2” genotype is spread across divergent habitats and geographical locations and the results of the analysis of surrounding haplotype SNPs suggested that the deletions occurred independently multiple times. Remarkably, 17 out of 25 relicts (accessions that inhabit ancestral habitat) [44], including one extremely divergent accession from the Cape Verde Islands likely possess “del-2” genotype. For two of them, Don-0 and Ped-0, we provided experimental verification. We also detected reciprocal duplications of the block of *AT3G18530*-*AT3G18535* genes (“dupl-2” genotype). The analysis of chromosome breakpoints performed in one-fourth of the accessions with “del-2” genotype (including relict Don-0) and in all but one accession with the “dupl-2” genotype (including Etna-2, an admixed accession of relict and non-relict) revealed that the events underlying structural rearrangements recurrently involved homology-based recombination between segmental duplications flanking the block of these two genes. In humans, the deletions/duplications between directly oriented LCRs show twofold genome-wide enrichment [53]. However, unlike disease-associated CNVs, which have been extensively studied in humans, the CNV described in the present study might represent a region under lower selective pressure (although this information needs further verification). Nevertheless, the precise localization of a genomic region highly prone to NAHR in the model plant Arabidopsis suggests some interesting possibilities regarding molecular studies on this phenomenon or targeted gene duplication.

NAHR is acknowledged to be the key mechanism leading to the formation of recurrent CNVs in animals and humans [24, 54, 55] and our molecular analysis (detection of reciprocal genotypes, breakpoints at LCR regions with no additional inserted sequence, sequence conversion events, random geographic distribution) provided strong evidence that *AT3G18530-AT3G18535* CNV was mediated by this mechanism, as well. It should be acknowledged, however, that it does not exclude the possibility that additional nonhomologous or microhomology-driven steps were involved in formation of the complex duplications at this site as well (exemplified by Etna-2, La-0 and Di-G accessions). In fact, extensive analysis of complex structural variations present in the human genomes revealed that in many cases, the template-switching mechanisms might contribute to their structural complexity [55, 56]. The third gene investigated in the present study, which also lies within mCNV, is *MSH2*. Although the previously described CNV_610 region [29] only partially covered the *MSH2* locus, the MLPA data obtained in the present study show that this duplication is larger (presumably affecting the entire gene). We observed the striking association of the *MSH2* copy number with the copy numbers of *AT3G18530* and *AT3G18535*. The *MSH2* duplication was not combined with the duplication of the remaining genes in only one case. In 11 accessions, all three of genes were duplicated, and in 7 accessions, the gene copy numbers were identical according to the ddPCR analysis. In 2 accessions, the *MSH2* copy number was lower (by 2 copies) and in 2 other accessions the *MSH2* copy number was higher (by 2 copies) than *AT3G18530*-*AT3G18535* (Additional file 2: Table S1). Additionally, high copy number alleles (8 copies and more) were exclusively observed among accessions with “dupl-3” genotypes. This finding suggests that the region harboring all three genes underwent duplications, likely involving molecular events different from those leading to the generation of the “dupl-2” and “del-2” genotypes. However, at present, we did not attempt to dissect these mechanisms or the chromosome breakpoints in accessions with the “dupl-3” genotype.

CNV may affect gene dosage and consequent phenotype [16]. *MSH2* is a conserved gene, essential for maintaining genome stability and preventing recombination events between non-identical sequences [57]. Mutations in this gene lead to microsatellite instability [58]. Moreover, *Atmsh2-1* knockout mutant lines rapidly accumulate mutations and show abnormalities in morphology and development, fertility, germination efficiency, seed/silique development, and seed set when propagated for 5 generations [59]. Consistent with these findings, none of the Arabidopsis accessions analyzed in the present study harbored *MSH2* deletions. Although evaluating the effect of the *MSH2* gene duplication at the transcription level was beyond the scope of the present study, we aim to highlight the potential impact of this variation. Future studies of accessions naturally varying according to *MSH2* copy number might enhance the functional characterization of the mismatch repair mechanisms in plants.

One issue concerning WGS-based CNV discovery is that different bioinformatics approaches might produce results (CNV lists) showing little overlap [1]. Therefore, the routine verification of new CNVs and the examination of the frequency of these changes in plant populations are needed before this new knowledge can be broadly applied to genome-wide association and agrigenomics studies. In humans, where CNV-genotyping is a routine task [60, 61], including the availability of commercial assays for some disease-related genes, multiple gene-overlapping CNVs have been studied in detail. No such standardized protocols exist for plants and the available CNV data are not abundant or preliminary. Previous reports regarding variation of Arabidopsis *MSH2*, *AT3G18530* and *AT3G18535* genes include CNV_610 and CNV_611 inferred by Cao et al. in Eurasian populations (Additional file 1: Figure S1) [29] and variation analysis among Swedish accessions, where two neighboring 3-kb duplicated regions were detected (variant Chr3:6375000 was present in FäL 1, Fäb-2, Tamm-2, TFÄ 06 and Västervik; variant Chr3:6372000 was present in FäL 1 and Fäb-2 only) [62]. Here we provided molecular evidence (MLPA, ddPCR and Sanger-based) that Tamm-2 harbors duplication of both *AT3G18530* and *AT3G18535* genes. Overall, the comprehensive information about a CNV status of these loci in Arabidopsis population may serve as a “gold standard” to validate genomewide tools for variant calling from WGS data.

In the present study, we compared two molecular approaches towards locus-specific CNV analysis that were optimized for usage in Arabidopsis: MLPA and ddPCR. We also designed sets of control probes and primers, localized in experimentally validated copy number stable regions, for genotyping any CNV of interest in Arabidopsis. MLPA is considered a gold standard in the molecular diagnosis of human diseases resulting from DNA copy number alterations [60]. Furthermore, MLPA has multiplexing ability and is cost-efficient when the analysis of hundreds or thousands of DNA samples is needed. Unlike ddPCR, which typically requires some optimization steps, MLPA is consistently performed under the same uniform conditions. However, the initial high cost of probe synthesis makes this approach suboptimal for CNV analysis in a smaller number of samples. In such cases, ddPCR would be much more time and cost-efficient, as this approach requires only standard PCR primers and is a one-step method, exhibiting exceptional sensitivity with less possibility for the introduction of errors [36, 63].

The discrete genotyping of mCNVs is a challenging task, and typically, the precise copy numbers could only be estimated for a lower number of copies (<6) [64–66]. Previously, we presented the robustness of the MLPA approach for the high-resolution genotyping of several disease-related mCNVs in humans, accurately genotyping integer copy numbers of up to 8 gene copies [41, 43]. More recently, ddPCR has been shown to provide accurate copy number assessments for mCNV in human populations, ranging from 0–15 copies [38]. Through direct comparison of the two approaches, we showed that ddPCR outperformed MLPA in discriminating high copy numbers. However, both methods produced highly consistent results and facilitated accurate copy number assessment across a common range of diploid copy numbers (0–8 copies). Moreover, both approaches are easy to apply for the systematic genotyping of gene CNV in plants and the validation of WGS data.

## Conclusions

Here we presented the first detailed, population-scale analysis of a complex CNV of a particular locus in Arabidopsis. We dissected the structure of a complex mCNV, spanning the *MSH2*, *AT3G18530* and *AT3G18535* genes in Arabidopsis genome, and evaluated the range and eco-geographical distribution of the copy numbers of these genes. The presented data provided insight into the mechanisms and factors driving CNV evolution in Arabidopsis and allowed us to create a model of recurrent duplications/deletions of *AT3G18530* and *AT3G18535* through homologous recombination between long repeats, flanking these genes. Our comprehensive case study provides foundation information for further analyses of CNV evolution in Arabidopsis and other plants, and their possible use in plant breeding. We also successfully applied two experimental approaches (MLPA and ddPCR) for CNV analysis in this plant. We directly compared the two techniques and showed their consistency in detecting DNA copy numbers up to 8. While MLPA is highly suitable for multiplexed analysis of several loci in a large number of samples, ddPCR should be the method of choice for discriminating loci with high copy numbers.

## Methods

### Plant materials

Arabidopsis seeds were obtained from NASC The European Arabidopsis Stock Centre. The seeds were surface-sterilized, vernalized for 3 days and subsequently planted on Jiffy pellets in ARASYSTEM containers (BETATECH). The plants were grown for 3 weeks in a growth chamber under long day conditions (16-h light; 8-h dark; 22 °C/18 °C, 70 % humidity), nourished with Murashige & Skoog medium, 0.5x (Serva). The leaves were frozen in liquid nitrogen and stored at −80 °C. Genomic DNA was extracted using the DNeasy Plant Mini Kit (Qiagen), followed by qualitative and quantitative evaluation on a Nanodrop 2000 spectrophotometer (Thermo Scientific) and with standard gel electrophoresis.

### MLPA probe set design and data analysis

The synthetic oligonucleotide half probes were designed as previously described [40, 41] and purchased (Integrated DNA Technologies). Each half probe comprised a target-specific sequence (23–29 nt each), the stuffer sequence of variable length and a universal primer sequence (Additional file 1: Table S2). Preferentially, target-specific sequences were localized in gene exons in SNP-free regions (based on a SNP map available for MPICao2010 data set [67]. The MLPA assays were performed using the SALSA MLPA Reagent Kit (MRC-Holland) according to the manufacturer’s guidelines, starting with 100 ng of genomic DNA and 1 pM of each half probe per reaction. The amplification products were separated through capillary electrophoresis on an ABI Prism 3130XL Genetic Analyzer (Applied Biosystems). The peak heights (signal intensities) were retrieved from the electropherograms using GeneMarker v.2.4.0 (SoftGenetics). For each sample, the peak heights were normalized to the average of the control probes. The low variation of the normalized values observed for probes ctrl1 to ctrl5 (SD <10 %) was used to verify the high quality of all MLPA assays. To facilitate data comparison across the probes and samples, the data are presented as a ratio to Col-0 accession. In 179 samples, the ratios for all control probes were between 0.8 and 1.2 (expected: 1.0) (Additional file 2: Table S1). In the remaining 10 samples, the ratio for only one control probe (ctrl4 or ctrl5, which generated DNA amplicons of the most extreme sizes in the Ath_CNV610-611_MLPA assay) showed only a small deviation from this range (min. observed ratio was 0.67 and max. was 1.38). Based on this analysis, the threshold values for deletion and duplication were set at 0.5 and 1.5, respectively.

### Droplet digital PCR assay design and analysis

The optimal sample concentration and primer annealing temperatures were evaluated in a series of test assays (0.05-50 ng DNA/well range and 56-60 °C temperature range tested, respectively, see Additional file 1: Figures S12 and S13). The final gene copy number assays were performed in 20-μl reactions containing 1× EvaGreen ddPCR Supermix (Bio-Rad), 200 nM gene specific primers (Additional file 1: Figure S2 and Table S3) and XbaI-digested DNA samples (2.5 ng). All XbaI restriction sites were located outside the predicted amplicons. Each reaction was mixed with 70 μl of Droplet Generation Oil (Bio-Rad), partitioned into approximately 18,000 droplets in a QX200 Droplet Generator (Bio-Rad), transferred to 96-well plates (Eppendorf) and sealed. The PCRs were performed using a C1000 Touch Thermal Cycler (Bio-Rad) with the following cycling conditions: 1× (95 °C for 5 min), 40× (95 °C for 30 s, 57 °C for 30 s, 72 °C for 45 s), 1× (4 °C for 5 min, 90 °C for 5 min) with 2 °C/s ramp rate. Immediately following end-point amplification, the fluorescence intensity of the individual droplets was measured using the QX200 Droplet Reader (Bio-Rad). The data analysis was performed using QuantaSoft droplet reader software (Bio-Rad). Positive and negative droplet populations were automatically detected. The template copy numbers [copies/μl PCR] were calculated using Poisson statistics and background-corrected based on the no-template control data. The absolute DNA copy numbers were subsequently obtained through within-sample normalization against data for the *DCL1* control gene. In all samples assayed, the calculated diploid copy number of the non-variable *HDA15* and *BRC1* genes flanking the analyzed CNV region was 2, confirming the accuracy of this normalization approach.

### CNV breakpoint detection and analysis

The genomic region covered through the Ath_CNV610-611_MLPA assay was divided into non-overlapping 1000-bp windows and subsequently BLASTN-searched against each other. The regions of significant similarity were further individually inspected and merged or extended to obtain final genomic coordinates of repeats. In accessions with the “del-2” CNV pattern, the regions of chromosome breakpoints were subsequently amplified using the following primers: forward: 5’-CCTAGAGCAGGAGTCGCAAG-3’ and reverse: 5’-CGCTTAAGTTAAGGAGATTTGACAACACCACAT-3.’ In accessions with the “dupl-2” CNV pattern, the regions of chromosome breakpoints were amplified using the following primers: left: 5’-GTGGGGGAGTTTGTGTCTCA-3’ and right: 5’- GTGGATTAAGGCGAATTCGACGACGAGATC-3.’ The amplifications were performed using PrimeSTAR GXL DNA Polymerase (Takara Bio), and the PCR products were resolved through agarose electrophoresis. For DNA sequencing, the PCR products were purified using the Clean & Concentrator Kit (Zymo) and sequenced on an ABI Prism 3130XL Genetic Analyzer (Applied Biosystems) using the Big Dye Terminator v.1.1 Cycle Sequencing Kit (Applied Biosystems).

### WGS-based variation analysis in 1001 genomes accessions

A list of SNPs in 20-kb genomic regions flanking the analyzed CNVs from each side (Chr3:6348000..6368000 and Chr3:6380000..6400000) was obtained from the 1001 Genomes Project server [67]. Out of 189 accessions used in this study the SNP data were available for 153 accessions. Additionally, Col-0 accession was included, with reference alleles in all positions. The positions were filtered to include only SNPs that were bi-allelic among 1,135 genomes. Subsequently, only SNPs of at least 10 % frequency among the subset of 154 analyzed accessions and with less than 20 % missing values per position were retained for the analysis. The SNPs were concatenated, aligned and imported to SplitsTree (version 4.14.4) in Nexus format (ready-to-use data file is included as Additional file 3). The phylogenetic network was then constructed using NeighborNet method [45]. LD analysis and R^2^ calculation was performed on the same data with the use of LDPlotter tool [68]. For the evaluation of “del-2” genotype prevalence, we obtained the sequences of *AT3G18530-AT3G18535* loci for 1,135 accessions using the Pseudogenome tool available in 1001 Genomes server.

## Additional files

Additional file 1:
**Figure S1.** Distribution of DNA copy number in regions covered by CNV_610 and CNV_611 in 80 natural accessions of Arabidopsis (MPICao2010 set). **Figure S2.** A schematic map of Arabidopsis genes covered by AthMSH2-MLPA assay. **Figure S3.** Exemplar electropherograms of AthMSH2-MLPA assay results. **Figure S4.** Pairwise correlation of MLPA signals obtained with probes mlpaB-mlpaG in AthMSH2-MLPA genotyping assay of Arabidopsis populations. **Figure S5.** Nonhierarchical phylogenetic network of a subset of 154 accessions based on 20-kb regions flanking the CNVs and its relation to the genetic groups defined by 1001 Genomes Consortium. **Figure S6.** Linkage disequilibrium (LD) at genomic regions surrounding the investigated CNVs. **Figure S7.** Rate of missing calls at *AT3G18530*-*AT3G18535* loci in pseudogenome sequences of 1135 Arabidopsis accessions. **Figure S8.** The sequence composition of the left and right breakpoints in accessions with “del-2” and “dupl-2” genotypes. **Figure S9.** Sequence alignment of CNV breakpoints in accessions with “del-2” genotype. **Figure S10.** Sequence alignment of CNV breakpoints in accessions with simple “dupl-2” genotype. **Figure S11.** Sequence alignment of CNV breakpoints in accessions with “dupl-2” genotype harboring extended duplication, that involves also the 3’ flank of the right LCR. **Figure S12.** Optimization of genomic DNA template input for ddPCR. **Figure S13.** Optimization of primer annealing temperatures for ddPCR. **Table S2.** Sequences of MLPA probes. **Table S3.** Gene specific primers used for ddPCR assays. (PDF 1563 kb)
Additional file 2:
**Table S1.** List of Arabidopsis accessions and gene copy numbers determined in MLPA- and ddPCR-based study. (XLSX 81 kb)
Additional file 3: Matrix data file with SNP data for 154 accessions, used for the construction of phylogenetic network. (TXT 16 kb)

## Abbreviations

CNV: Copy number variation
ddPCR: Droplet digital PCR
dHj: Double Holliday junction
DSB: Double strand break
EPSP: 5-enolpyruvylshikimate 3-phosphate
LCR: Low copy repeat
LD: Linkage disequilibrium
mCNV: Multiallelic CNV
MLPA: Multiplex ligation-dependent probe amplification
NAHR: Non-allelic homologous recombination
SCN: Soybean cyst nematode
WGS: Whole-genome sequencing
