# Supplementary material for: Arabidopsis thaliana population analysis reveals high plasticity of the genomic region spanning MSH2, AT3G18530 and AT3G18535 genes and provides evidence for NAHR-driven recurrent CNV events occurring in this location
Source: BMC Genomics. 2016 Nov 8;17:893. doi: 10.1186/s12864-016-3221-1 (PMC5101643; doi:10.1186/s12864-016-3221-1)
Supplement: Additional file 1: — Figure S1. Distribution of DNA copy number in regions covered by CNV_610 and CNV_611 in 80 natural accessions of Arabidopsis (MPICao2010 set). Figure S2. A schematic map of Arabidopsis genes covered by AthMSH2-MLPA assay. Figure S3. Exemplar electropherograms of AthMSH2-MLPA assay results. Figure S4. Pairwise correlation of MLPA signals obtained with probes mlpaB-mlpaG in AthMSH2-MLPA genotyping assay of Arabidopsis populations. Figure S5. Nonhierarchical phylogenetic network of a subset of 154 accessions based on 20-kb regions flanking the CNVs and its relation to the genetic groups defined by 1001 Genomes Consortium. Figure S6. Linkage disequilibrium (LD) at genomic regions surrounding the investigated CNVs. Figure S7. Rate of missing calls at AT3G18530-AT3G18535 loci in pseudogenome sequences of 1135 Arabidopsis accessions. Figure S8. The sequence composition of the left and right breakpoints in accessions with “del-2” and “dupl-2” genotypes. Figure S9. Sequence alignment of CNV breakpoints in accessions with “del-2” genotype. Figure S10. Sequence alignment of CNV breakpoints in accessions with simple “dupl-2” genotype. Figure S11. Sequence alignment of CNV breakpoints in accessions with “dupl-2” genotype harboring extended duplication, that involves also the 3’ flank of the right LCR. Figure S12. Optimization of genomic DNA template input for ddPCR. Figure S13. Optimization of primer annealing temperatures for ddPCR. Table S2. Sequences of MLPA probes. Table S3. Gene specific primers used for ddPCR assays. (PDF 1563 kb) [file 12864_2016_3221_MOESM1_ESM.pdf]

Additional File 1:

Figure S1 Distribution of DNA copy number in regions covered by CNV_610 and CNV_611 in 80 natural accessions of Arabidopsis (MPICao2010 set).

Figure S2. A schematic map of Arabidopsis genes covered by AthMSH2-MLPA assay**.**

Figure S3 Exemplar electropherograms of AthMSH2-MLPA assay results.

Figure S4 Pairwise correlation of MLPA signals obtained with probes mlpaB-mlpaG in AthMSH2-MLPA genotyping assay of Arabidopsis populations.

Figure S5 Nonhierarchical phylogenetic network of a subset of 154 accessions based on 20-kb regions flanking the CNVs and its relation to the genetic groups defined by 1001 Genomes Consortium.

Figure S6 Linkage disequilibrium (LD) at genomic regions surrounding the investigated CNVs.

Figure S7 Rate of missing calls at *AT3G18530-AT3G18535* loci in pseudogenome sequences of 1135 Arabidopsis accessions.

Figure S8 The sequence composition of the left and right breakpoints in accessions with “del-2” and “dupl-2” genotypes.

Figure S9 Sequence alignment of CNV breakpoints in accessions with “del-2” genotype.

Figure S10 Sequence alignment of CNV breakpoints in accessions with simple “dupl-2” genotype.

Figure S11 Sequence alignment of CNV breakpoints in accessions with “dupl-2” genotype harboring extended duplication, that involves also the 3’ flank of the right LCR.

Figure S12 Optimization of genomic DNA template input for ddPCR.

Figure S13 Optimization of primer annealing temperatures for ddPCR.

Table S2 Sequences of MLPA probes.

Table S3 Gene specific primers used for ddPCR assays.

Fig. S1 Distribution of DNA copy number in regions covered by CNV_610 and CNV_611 in 80 natural accessions of *A. thaliana* (MPICao2010 set). The source data are from [29]. DNA copy number is presented as relative to the reference genome, Col-0. The division according to the geographic origin of the accessions is identical to that proposed in the original paper.

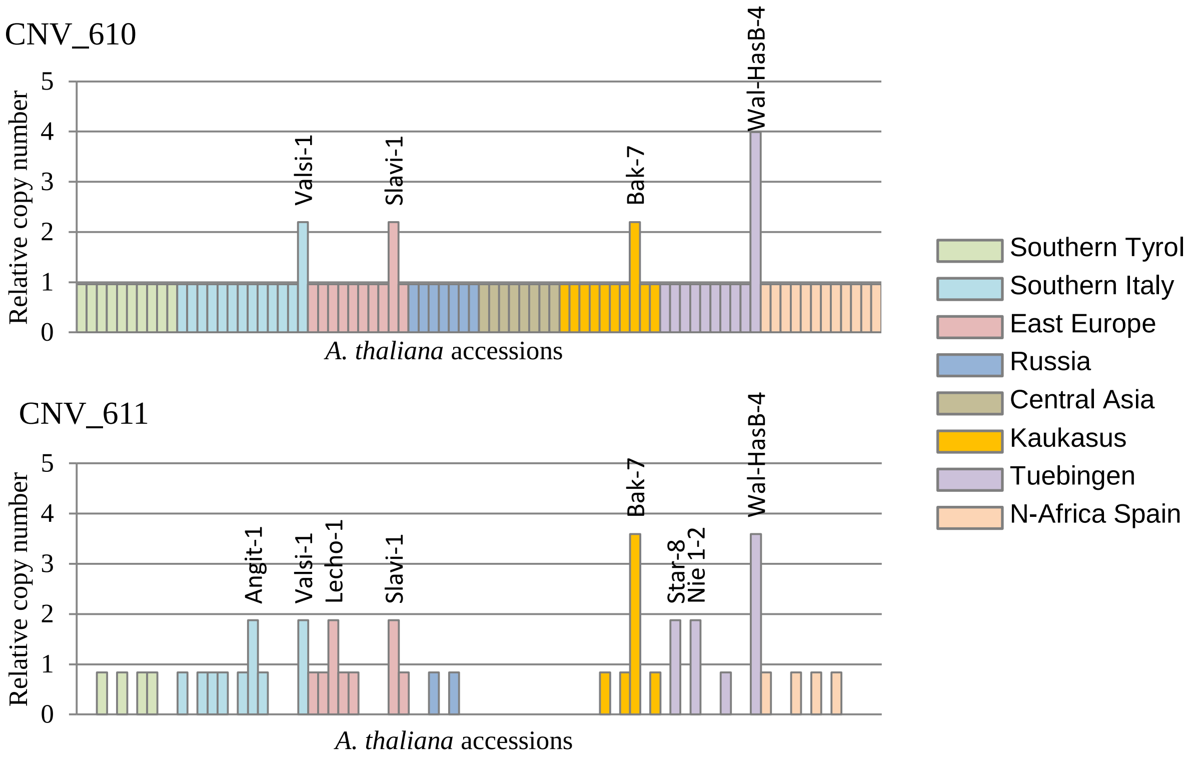

Fig. S2 A schematic map of *A. thaliana* genes covered by AthMSH2-MLPA assay. Approximate positions of MLPA probes are indicated over the gene models. For genes with alternative splicing isoforms (*HDA15*, *BRC1*, *DCL1*, *AT4G21580*) the longest gene model is presented and the probes are localized in conserved exons. Blue rectangles represent exons (dark for coding sequence, light for untranslated regions); blue lines represent introns; arrows show the gene orientation on genomic DNA reference sequence. Green boxes indicate the overlap of CNV regions inferred by WGS data analysis [29]. Approximate positions of primer pairs used for ddPCR-based verification are indicated below the gene models.

AT3G18520 (HDA15)

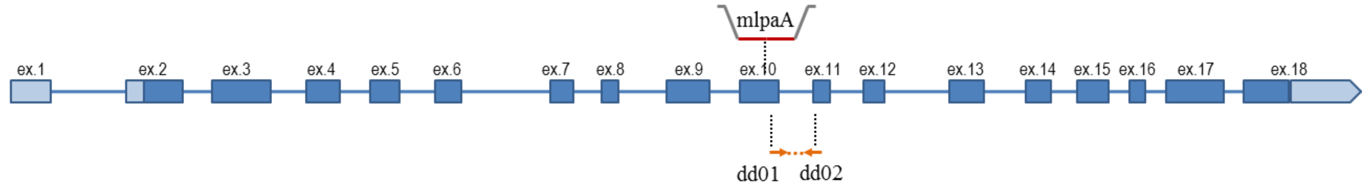

AT3G18524 (MSH2)

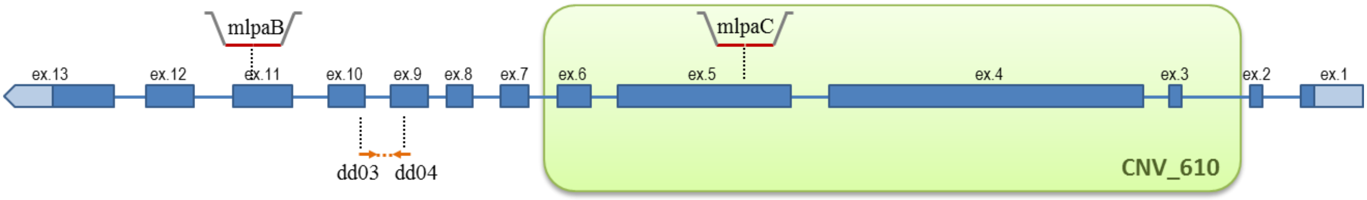

AT3G18530; *AT3G18535*

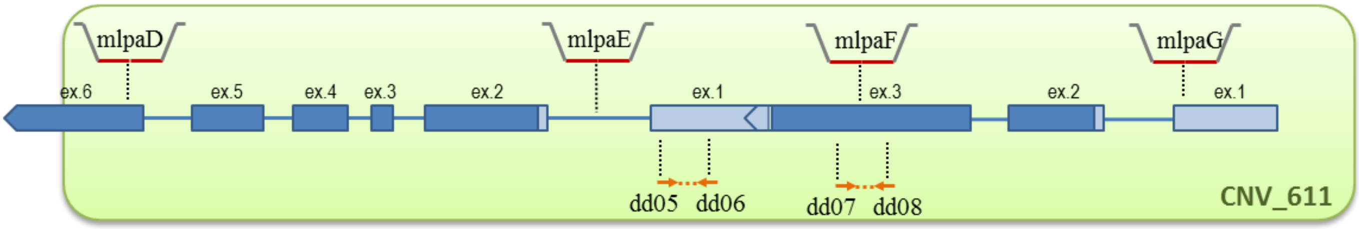

AT3G18550 (BRC1)

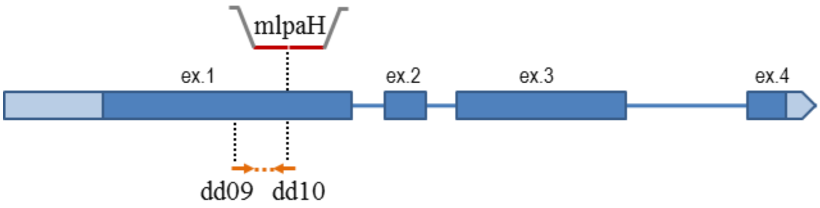

AT1G01040 (DCL1)

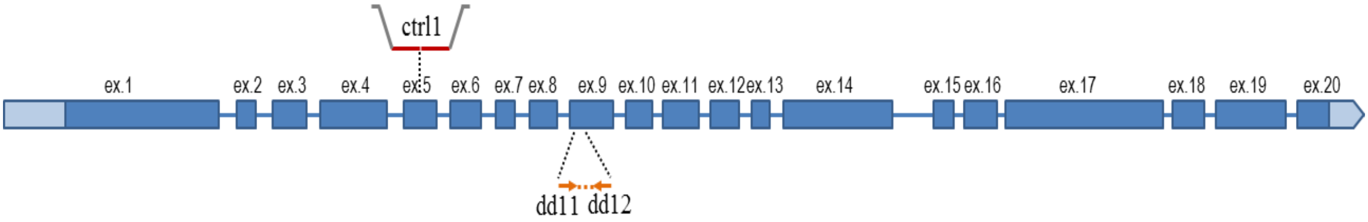

AT4G21580

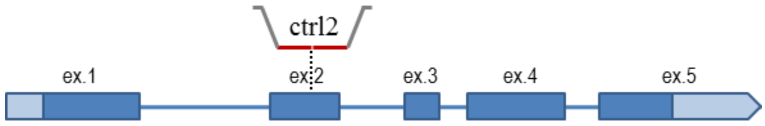

AT2G36230 (APG10)

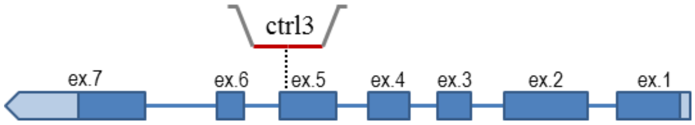

AT5G23290 (PFD5)

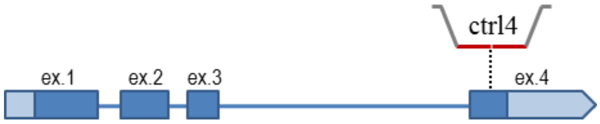

AT1G73010 (PS2)

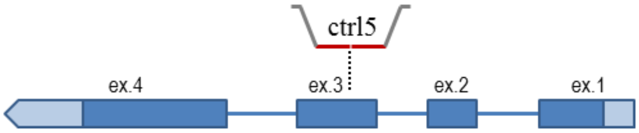

Fig. S3 Exemplar electropherograms of AthMSH2-MLPA assay results. The data are representative for accessions with: **A** – no copy number changes detected; **B** - amplification of *MSH2*, *AT3G18530* and *AT3G18535* genes; **C** – deletion of *AT3G18530* and *AT3G18535* genes. Normalized peak height from the Gene Marker v.2.4.0 analysis are presented (note that the scale on electropherogram B is different from A and C). Probes' IDs (see Table 1 for detailed description) are shown under the electropherograms. Colors indicate target genes: *MSH2* (green) *AT3G18530* (red) *AT3G18535* (blue), *HDA15* (brown), *BRC1* (purple). All control probes are in black. DNA fragment sizes are indicated on the upper scale. The data presented on the Figure were obtained for Col-0 (A), Wal-HasB-4 (B) and Voeran-1 (C) accessions.

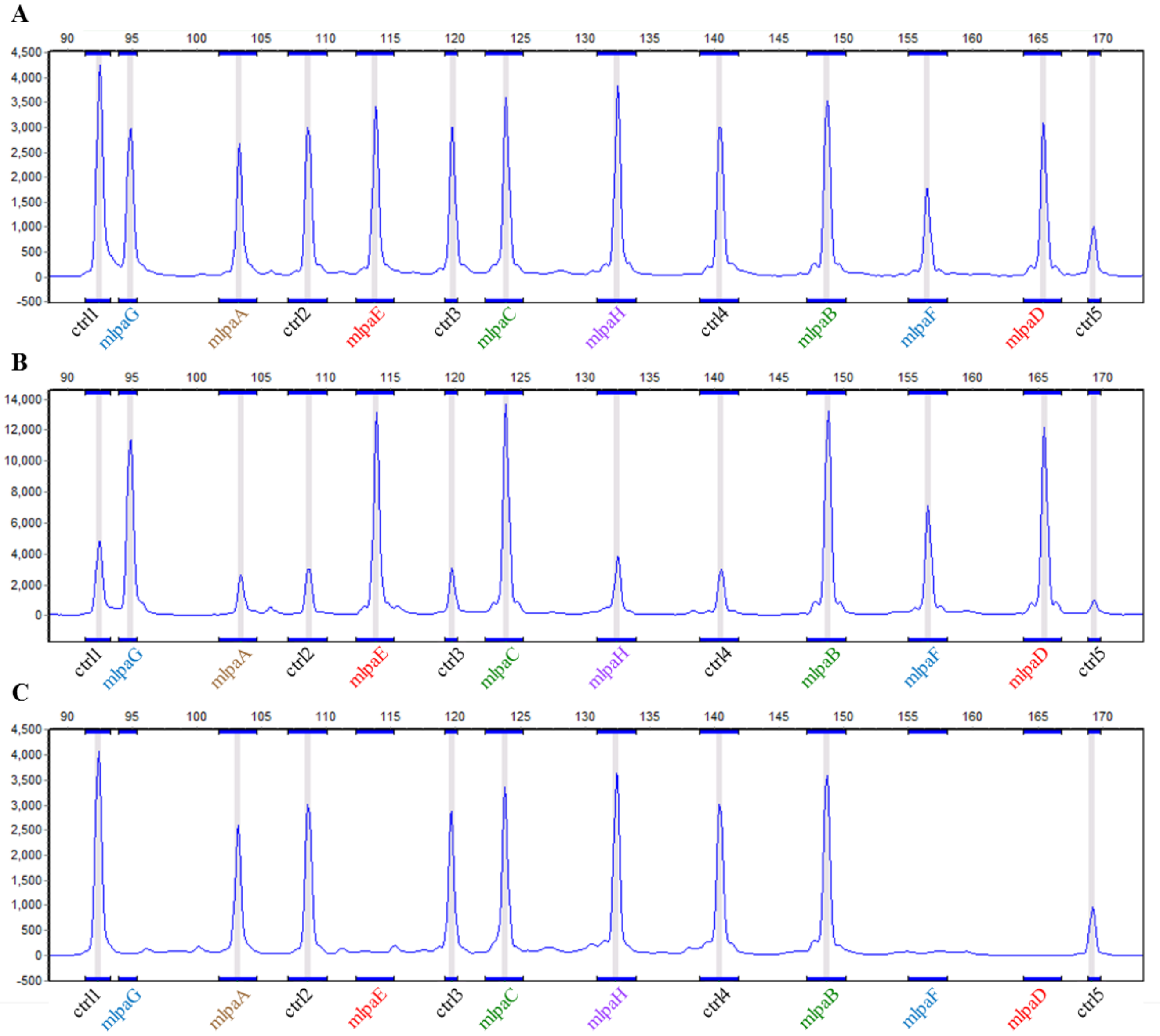

Fig. S4 Pairwise correlation of MLPA signals obtained with probes mlpaB-mlpaG in AthMSH2-MLPA genotyping assay of Arabidopsis populations. R^2 – the square of the Pearson linear regression coefficient

MSH2		*AT3G18530*		*AT3G18535*		
mlpaB	mlpaC	mlpaD	mlpaE	mlpaF	mlpaG	***R^2***
**1.000**	0.988	0.472	0.439	0.460	0.390	mlpaB
	**1.000**	0.532	0.507	0.525	0.458	mlpaC
		**1.000**	0.957	0.968	0.938	mlpaD
			**1.000**	0.994	0.991	mlpaE
				**1.000**	0.982	mlpaF
					**1.000**	mlpaG

Fig. S5 Nonhierarchical phylogenetic network of a subset of 154 accessions (including Col-0) based on 20-kb regions flanking the CNVs and its relation to the genetic groups defined by 1001 Genomes Consortium. The Network was constructed with the NeighborNet algorithm, based on bi-allelic SNPs of at least 10% frequency, surrounding the CNV region from both sides (20-kb flanks). Each accession is marked with a symbol shape indicating its CNV pattern and with a color indicating the genetic group it belongs to, according to a recent population study of 1135 Arabidopsis accessions [44]. No clear evolutionary splits between the accessions harboring distinct copy number of *MSH2*, *AT3G18530* and *AT3G18535* genes could be observed (see main text for details).

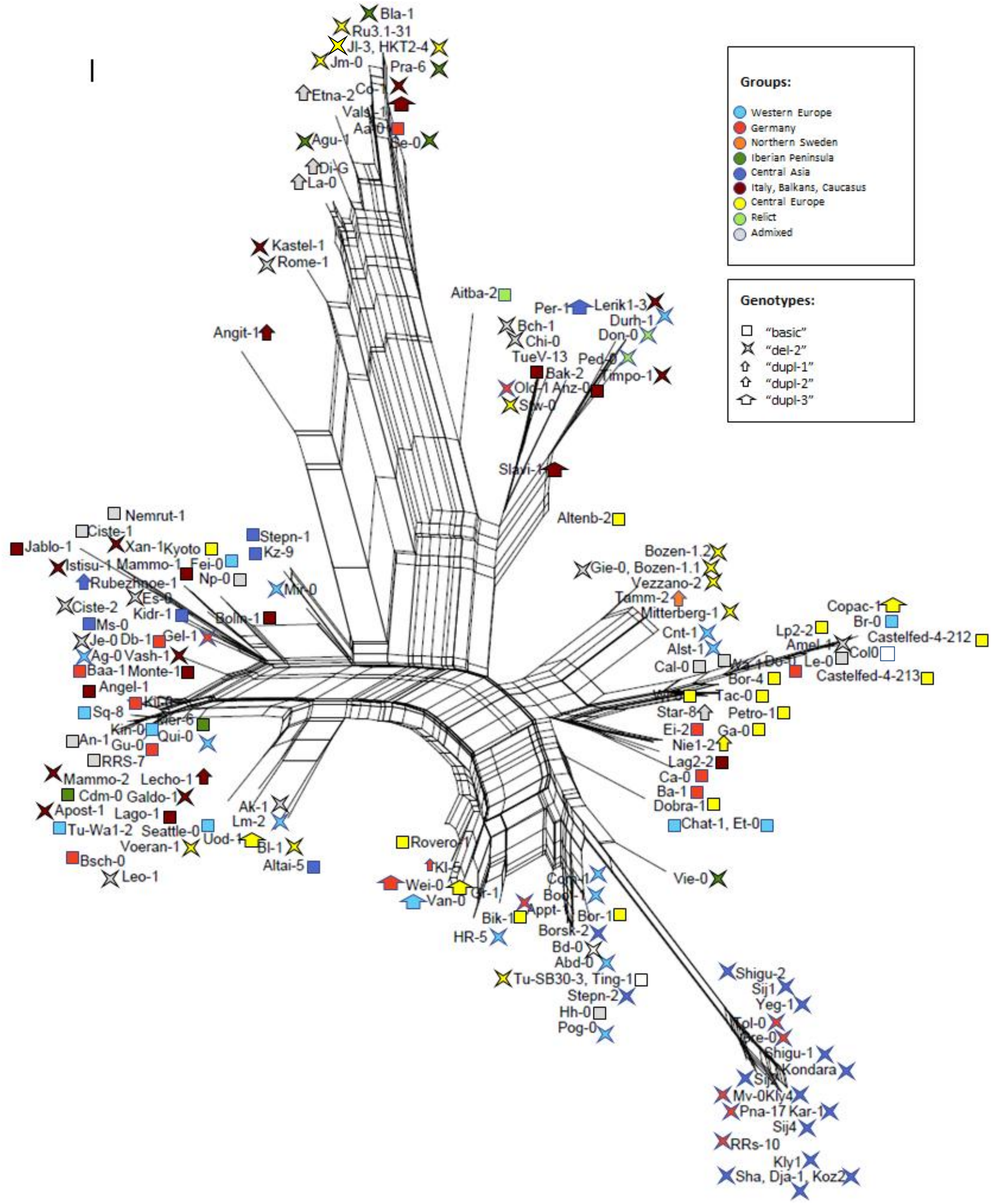

Fig. S6 Linkage disequilibrium (LD) at genomic regions surrounding the investigated CNV. LD plot shows correlation between the pairs of bi-allelic SNPs in the 20-kb DNA regions which flank *MSH2-AT3G18530-AT3G18535* genes, in 154 accessions (including Col-0) as well as the correlation between the SNPs and the CNV patterns. The SNPs are mapped to their physical positions along the chromosome 3 (top) with black lines. Solid green arrows indicate genes; the yellow rectangle highlights *MSH2, AT3G18530* and *AT3G18535* genes. CNV genotypes were incorporated into the analysis as TRUE or FALSE values (i.e. the accession harbors/not harbors the indicated genotype, respectively; "dupl-1" genotype was excluded from this analysis). Although LD blocks were detected in the analyzed region from each side of CNV, we observed no correlation between any CNV pattern and any SNP ($R^2 < 0.3$, data highlighted by the red frame).

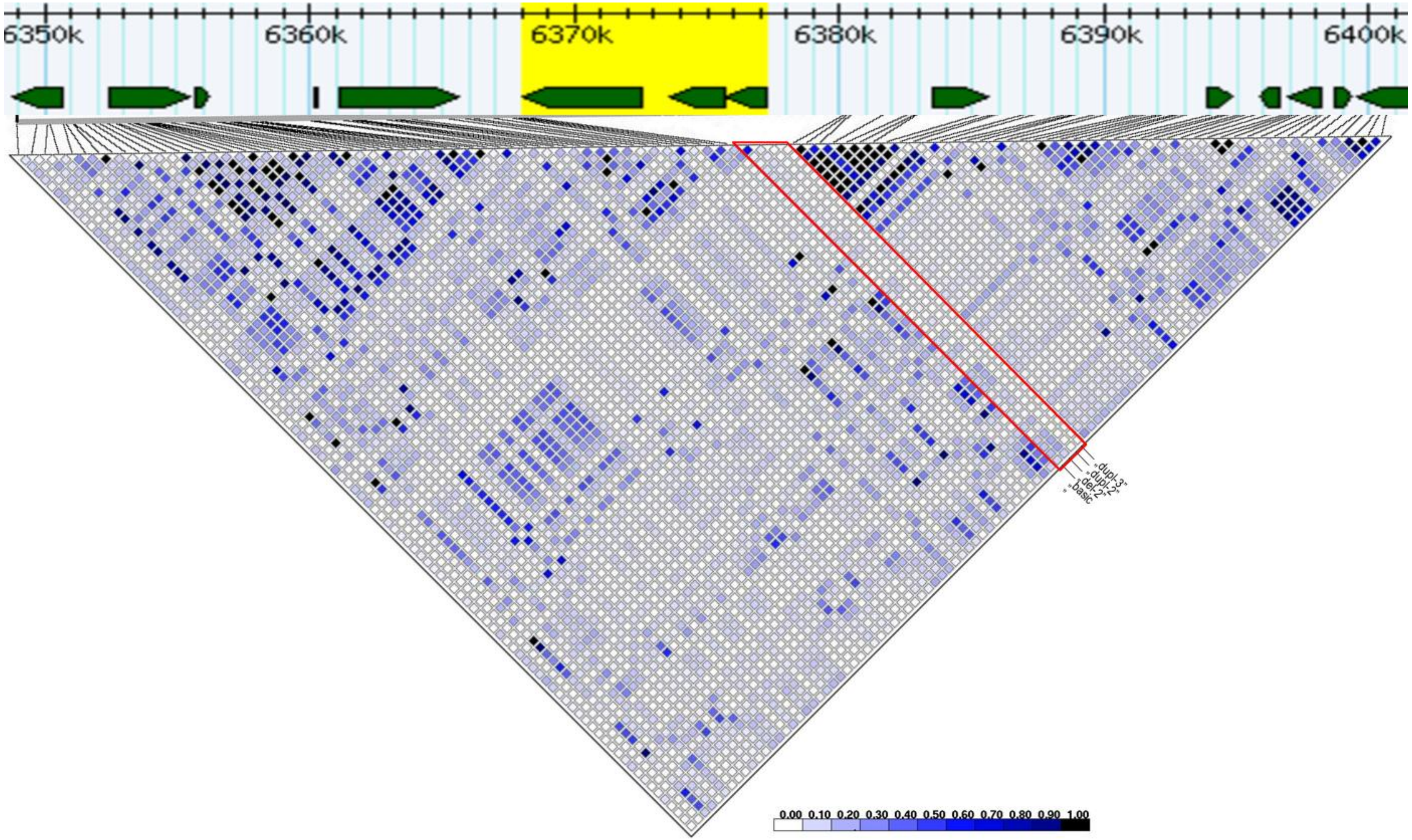

Fig. S7 Rate of missing calls at *AT3G18530-AT3G18535* loci in pseudogenome sequences of 1135 Arabidopsis accessions.

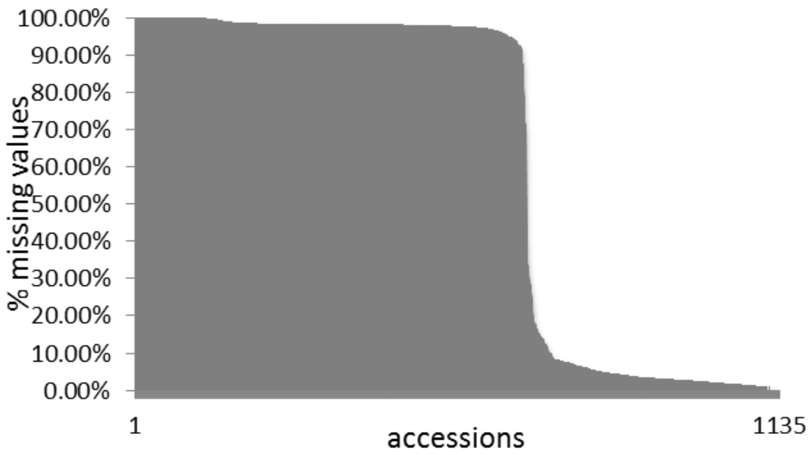

Fig. S8 The sequence composition of the left and right breakpoints in accessions with "del-2" and "dupl-2" genotypes. A – "basic" genotype. The 9-nt sequence (TGAACAAGG) localized at the 3' end of the left LCR shows homology (1 mismatch) to the sequence located downstream of the right LCR (TGAATAAGG); **B** – the "del-2" genotype; **B** – the simple "dupl-2" genotype, observed in Nie 1-2, Star-8, Tamm-2, Lecho-1 and Rubezhnoe-1 accessions; **D** – the "dupl-2" genotype with extended duplication region, observed in La-0, Etna-2 and Di-G accessions, with the indication of additional, microhomology-based sequence template switching between the 9-nt sequences (the recombined sequence is underlined). The sequence alignments of the regions marked by dashed-line boxes are presented in Supplementary Figs. S9-S11.

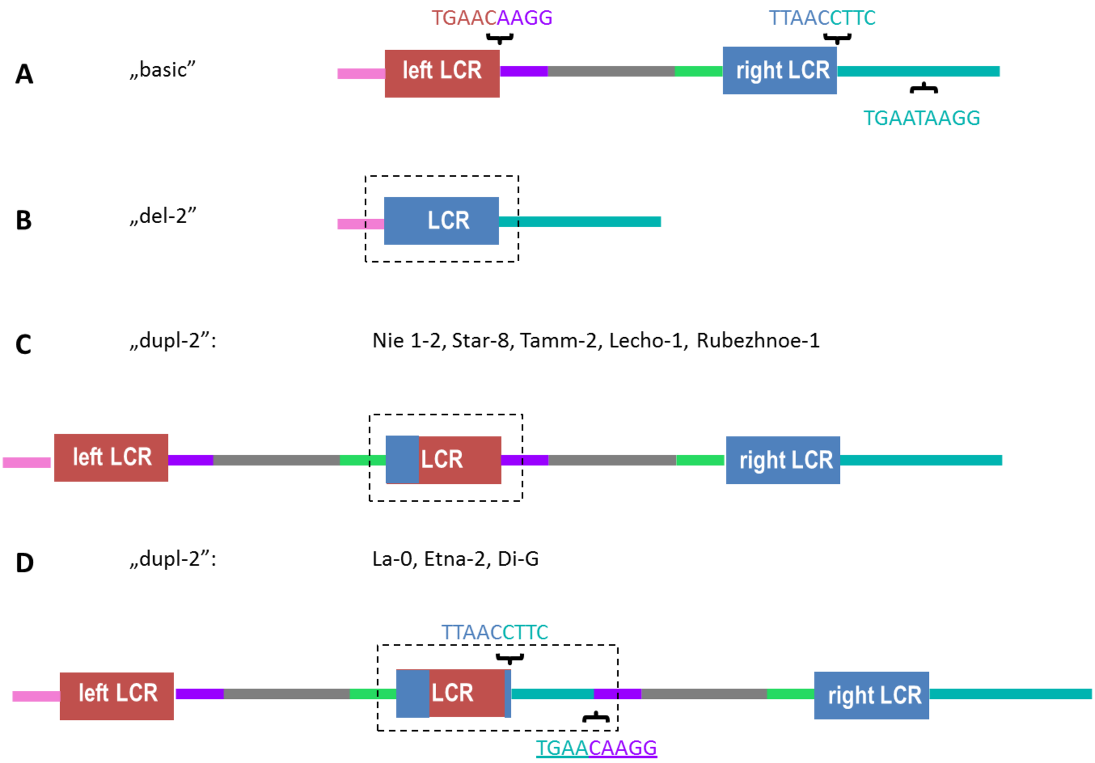

On next pages:

Fig. S9 Sequence alignment of CNV breakpoints in accessions with "del-2" genotype. The sequences are aligned along with the LCRs. Left LCR (Chr3:6372413..6373650) is presented with its 38 nt of its 5' flanking region (highlighted in pink) and right LCR (Chr3:6377368..6378605) is presented with its with 114 nt of its 3' flanking region (highlighted in teal). The colors of LCR flanking regions match those in Supplementary Fig. S8. Positions distinguishing both LCRs are highlighted by yellow boxes.

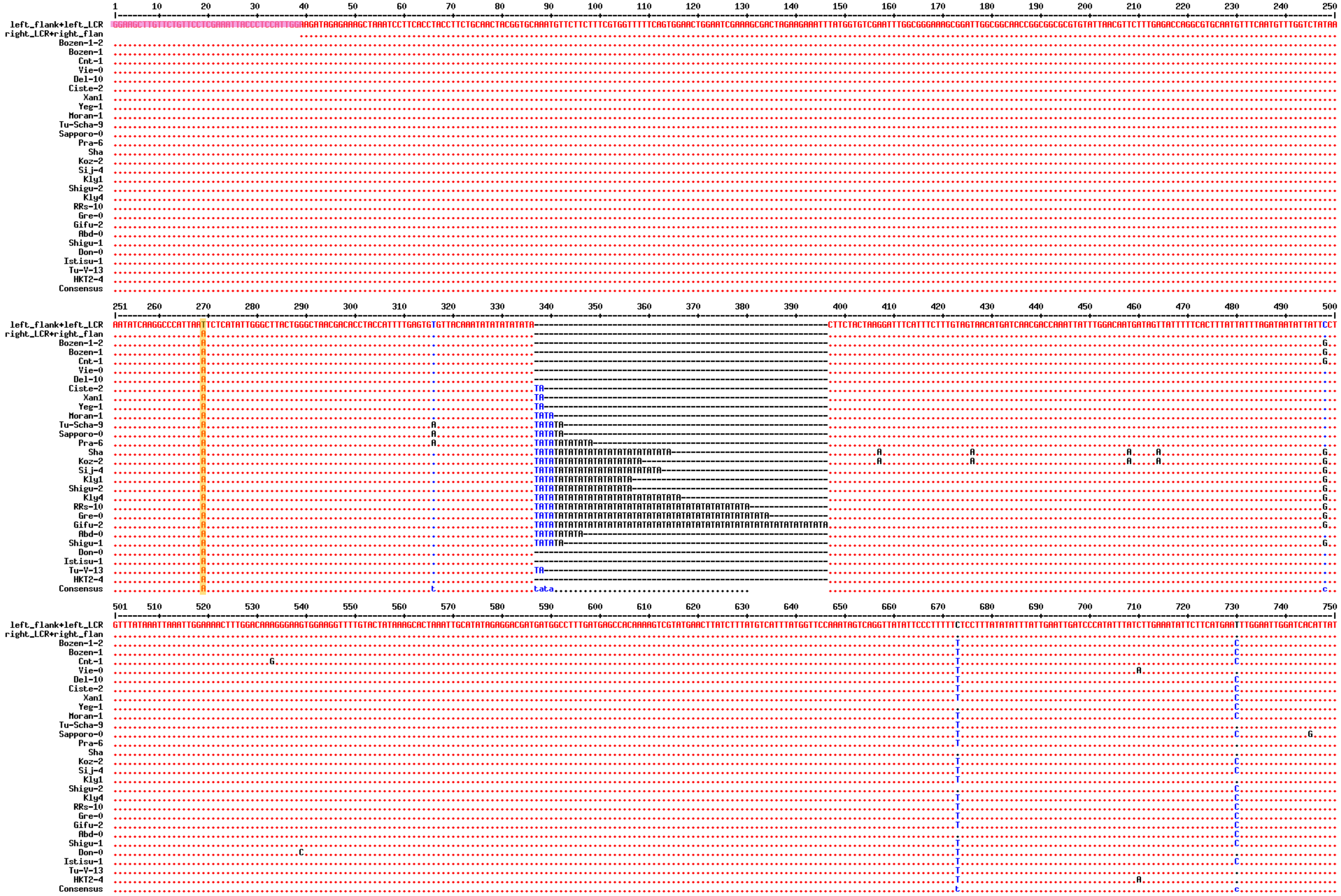

left_flank+left_LCR
right_LCR+right_flan
Bozen-1-2
Bozen-1
Cnt-1
Vie-0
Del-10
Ciste-2
Xan1
Yeg-1
Moran-1
Tu-Scha-9
Sapporo-0
Pra-6
Sha
Koz-2
Sij-4
Kly1
Shigu-2
Kly4
RRs-10
Gre-0
Gifu-2
Abd-0
Shigu-1
Don-0
Istisu-1
Tu-V-13
HKT2-4
Consensus

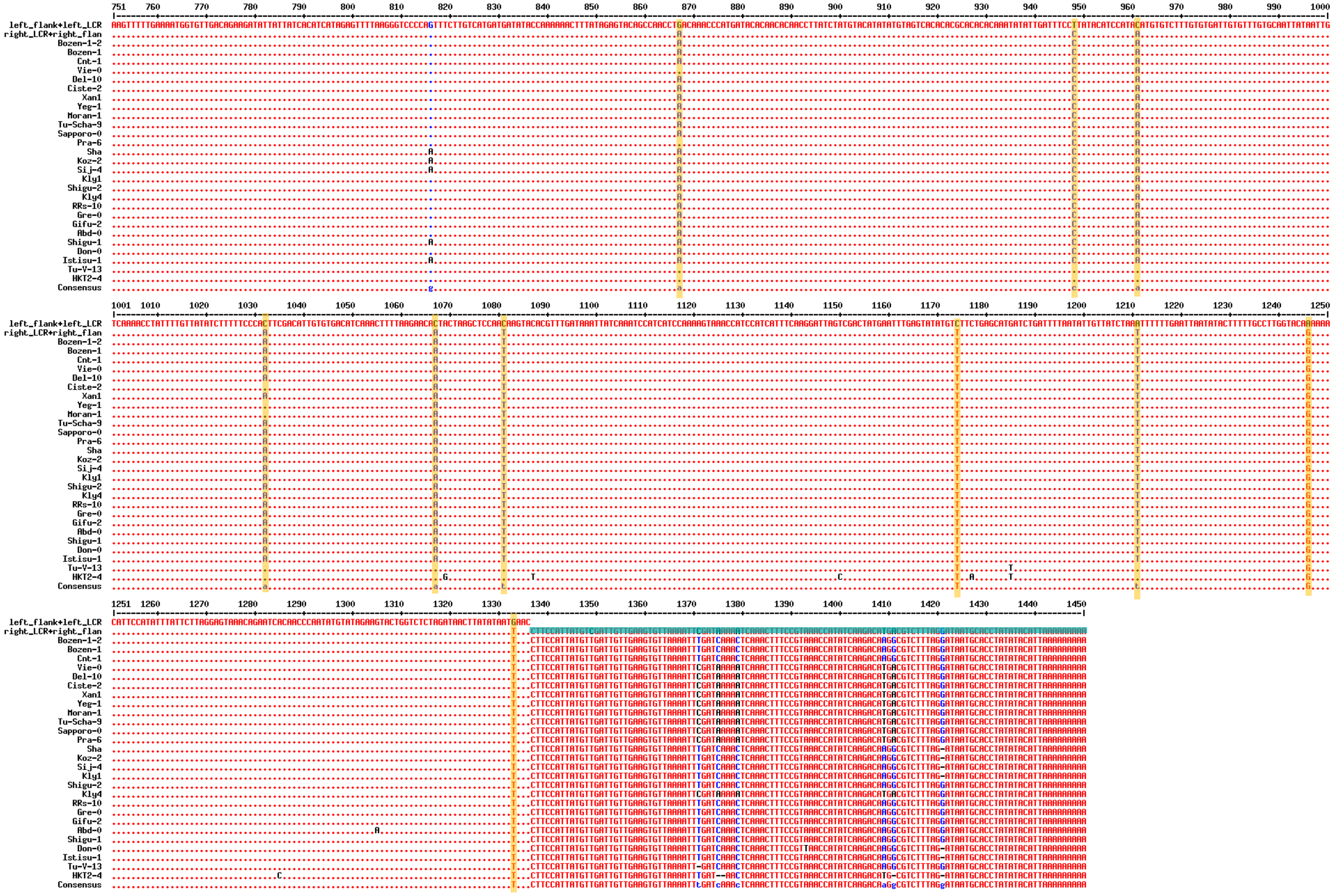
left_flank+left_LCR
right_LCR+right_flan
Bozen-1-2
Bozen-1
Cnt-1
Vie-0
Del-10
Ciste-2
Xan1
Yeg-1
Moran-1
Tu-Scha-9
Sapporo-0
Pra-6
Sha
Koz-2
Sij-4
Kly1
Shigu-2
Kly4
RRs-10
Gre-0
Gifu-2
Abd-0
Shigu-1
Don-0
Istisu-1
Tu-V-13
HKT2-4
Consensus

On next page:

Fig. S10 Sequence alignment of CNV breakpoints in accessions with simple "dupl-2" genotype. The sequences are aligned along with the LCRs. Left LCR (Chr3:6372413..6373650) is presented with its 3' flanking region (highlighted in purple) and right LCR (Chr3:6377368..6378605) is presented with its with 5' flanking region (highlighted in green). The colors of LCR flanking regions match those in Supplementary Fig. S8. Positions distinguishing both LCRs are highlighted by yellow boxes.

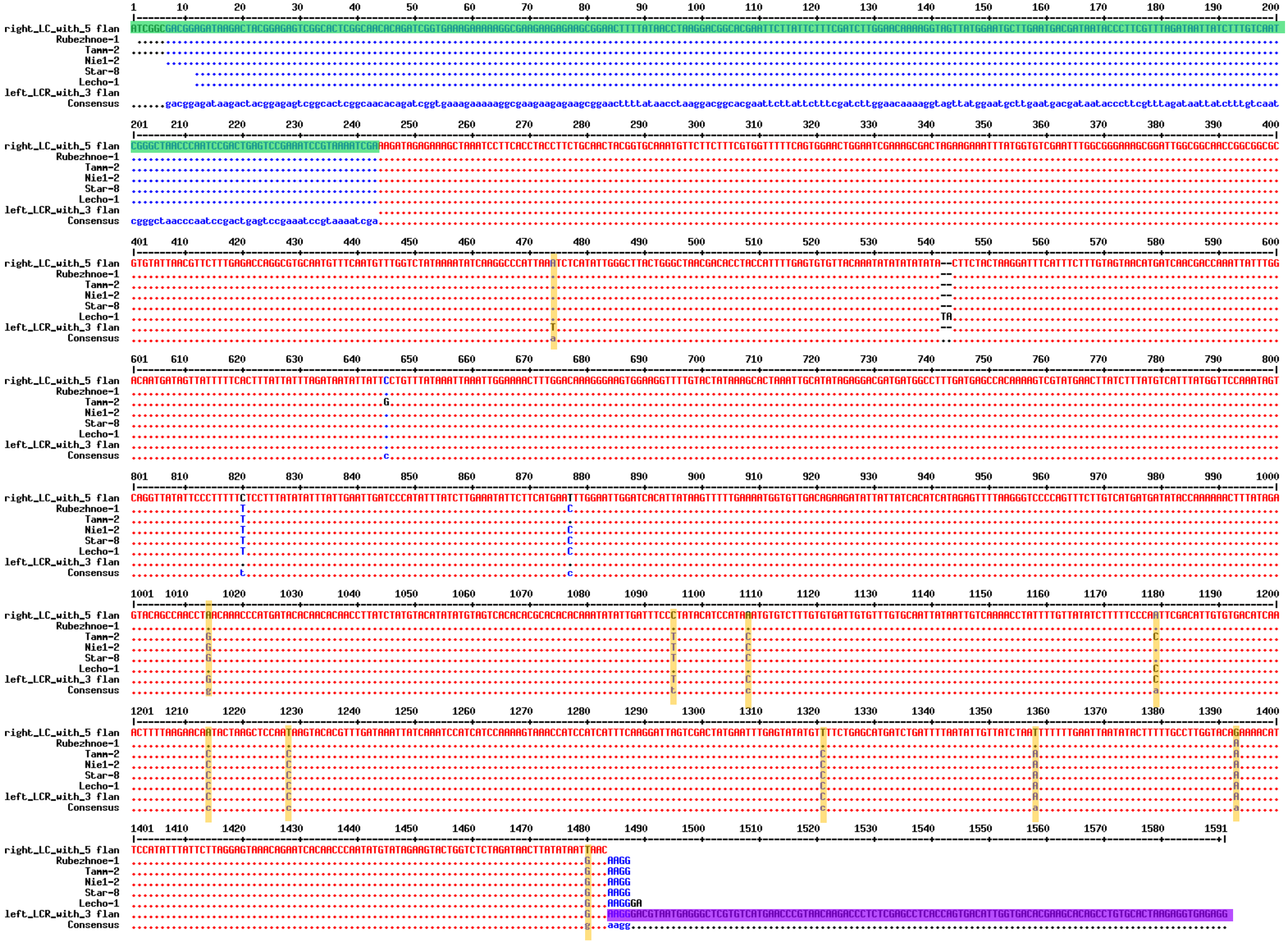
right_LC_with_5 flan
Rubezhnoe-1
Tamm-2
Niel-2
Star-8
Lecho-1
left_LCR_with_3 flan
Consensus

On next pages:

Fig. S11 Sequence alignment of CNV breakpoints in accessions with "dupl-2" genotype harboring extended duplication, that involves also the 3' flank of the right LCR. The sequences are aligned along with the LCRs. Left LCR (Chr3:6372413..6373650) is presented with its 3' flanking region (highlighted in purple) while right LCR (Chr3:6377368..6378605) is presented with both its with 5' flanking region (highlighted in green) and 3' flanking region (highlighted in teal). Positions distinguishing both LCRs are highlighted by yellow boxes, except for the last differentiating position, which is highlighted in orange. This position is within the microhomology region, which mediated the template switching in Di-G Etna-2 and La-0 accessions. The colors of LCR flanking regions match those in Supplementary Fig. S8.

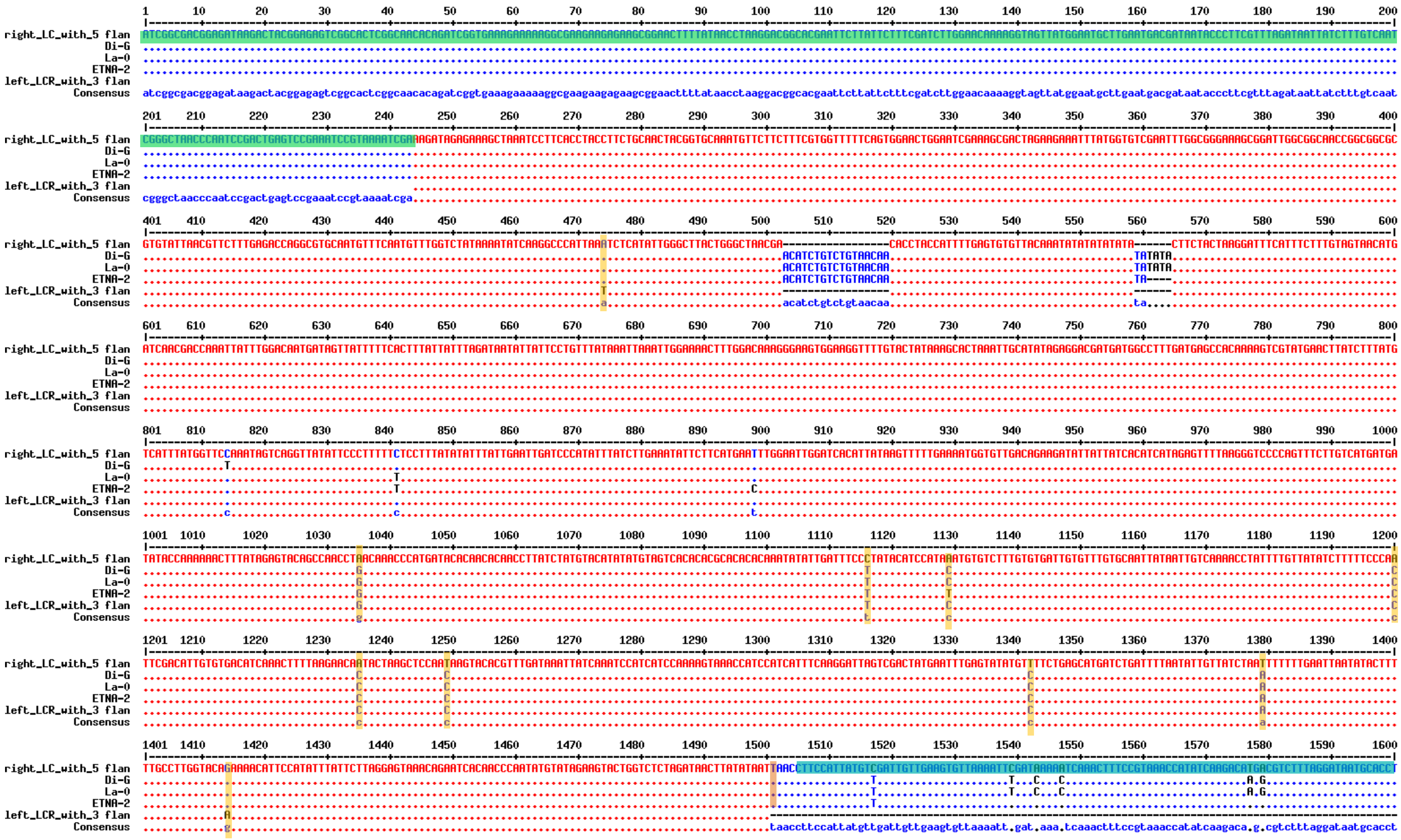
right_LC_with_5 flan
Di-G
La-0
ETNA-2
left_LCR_with_3 flan
Consensus

```
                   1601     1610      1620      1630      1640      1650      1660      1670      1680      1690      1700      1710      1720      1730      1740      1750      1760      1770      1780      1790      1800
                   |--------+---------+---------+---------+---------+---------+---------+---------+---------+---------+---------+---------+---------+---------+---------+---------+---------+---------+---------+---------|
right_LC_with_5 flan ATATACATTAAAAAAAAATGGCACAGAAATAAGATGTACGTTTATATGTACAACAACCTATTACATCATAAACCATAT----TTTAGTGGTGAAGCATGGAACAACAAACTTAGTAACTTGGACATTATTTGAGTGTTGTCGAAGACTCGAAGCCATTTATAAAGGTTTTAAATGATTAAAAACAAAA---AAAAACGAA
                Di-G ..............................................................................----.......................................................................................................CG.---.....AA..
                La-0 ..............................................................................ATAT..........................................................................................................GCA.....AA..
              ETNA-2 .................-............................................................ATAT..........................................................................................................GC-.....AA..
left_LCR_with_3 flan --------------------------------------------------------------------------------------------------------------------------------------------------------------------------------------------------------
           Consensus atatacattaaaaaaaaatggcacagaaataagatgtacgtttatatgtacaacaacctattacatcataaaccatat....tttagtggtgaagcatggaacaacaaacttagtaacttggacattatttgagtgttgtcgaagactcgaagccatttataaaggttttaaatgattaaaaacaaaa...aaaaaaaaa

                   1801     1810      1820      1830      1840      1850      1860      1870      1880      1890      1900      1910      1920      1930      1940      1950      1960      1970      1980      1990      2000
                   |--------+---------+---------+---------+---------+---------+---------+---------+---------+---------+---------+---------+---------+---------+---------+---------+---------+---------+---------+---------|
right_LC_with_5 flan AAAAAAAGAGCATCAAGATGTTACATCGTCAGGGAAAAGAACATCAATTTCTAAGTAAACTCGTAAAATTAAGGTTGAAATGCAGACATTGATAATGTAGGATTGGAAATATATTTTTTCAAAAAATAAGCAATTTATAATCATTTCATATATATATGAGAAGCTGAGCAATAAGAAAAAAAGACGTGAGAAAGACCATT
                Di-G ..............................G..........................................................C.................................................................................C............................
                La-0 ..............................G..........................................................C.................................................................................C............................
              ETNA-2 ..............................G..........................................................C.................................................................................C............................
left_LCR_with_3 flan --------------------------------------------------------------------------------------------------------------------------------------------------------------------------------------------------------
           Consensus aaaaaaagagcatcaagatgttacatcgtcggggaaaagaacatcaatttctaagtaaactcgtaaaattaaggttgaaatgcagacatcgataatgtaggattggaaatatattttttcaaaaaataagcaatttataatcatttcatatatatatgagaagctgagcaacaagaaaaaaagacgtgagaaagaccatt

                   2001     2010      2020      2030      2040      2050      2060      2070      2080      2090      2100      2110      2120      2130      2140      2150      2160      2170      2180      2190      2200
                   |--------+---------+---------+---------+---------+---------+---------+---------+---------+---------+---------+---------+---------+---------+---------+---------+---------+---------+---------+---------|
right_LC_with_5 flan AAAATAATCAACTGGATAAAACTATTCAGTTATTAGATGACTGATATGAATAATAAGTTCTTCGTAAATGTGGAGTTCTAATAGTAGAAATTAAGTGAAGTATATGACTTTGTTGCCCAAAAAAATGAAAAAAAAAACTAG-TATATATATATGACTTTTATCCTCTTACATTTCGCACCATATAGTGGCTTCAAGTATT
                Di-G .....................................................................T............................C.....C....--..............G........CT.G..TA.........--...............................................
                La-0 .....................................................................T............................C.....C....--..............G........CT.G..TA.........--...............................................
              ETNA-2 .....................................................................T............................C.....C....--..............G........CT.G..TA..........................................................
left_LCR_with_3 flan --------------------------------------------------------------------------------------------------------------------------------------------------------------------------------------------------------
           Consensus aaaataatcaactggataaaactattcagttattagatgactgatatgaataataagttcttcgtaaatttggagttctaatagtagaaattaagtgacgtatacgact..gttgcccaaaaaaaggaaaaaaactagtatatatatatat..gacttttatcctcttacatttcgcaccatatagtggcttcaagtatt

                   2201     2210      2220      2230      2240      2250      2260      2270      2280      2290      2300      2310      2320      2330      2340      2350      2360      237072
                   |--------+---------+---------+---------+---------+---------+---------+---------+---------+---------+---------+---------+---------+---------+---------+---------+---------+--------+-|
right_LC_with_5 flan TTCCTTTTTCTGAAGTTTTTCCCACCAAGCTAAAACATCATTTTCTGAAGTTACAATATTGAATAAGGTATAATTTGAAGACA
                Di-G ...............................................................C....G.CGTAA...G.G.TCGTGTCATGAACCCGTAACAAGACCCTCTCGAGCCTCACCAGTGACATTGGTGACACGAAGCACAGCCTGTGCA
                La-0 ...............................................................C....G.CGTAA...G.G.TCGTGTCATGAACCCGTAACAAGACCCTCTCGAGCCTCACCAGTGACATTGGTGACACGAAGCACAGCCTGTGCACTAAGAG
              ETNA-2 ...............................................................C....G.CGTAA...G.G.TCGTGTCATGAACCCGTAACAAGACCCTCTCGAGCCTCACCAGTGACATTGGTGACACGAAGCACAGCCTGTGCACTAAGAGGTGAGAG
left_LCR_with_3 flan ------------------------------------------------------------...C....G.CGTAA...G.G.TCGTGTCATGAACCCGTAACAAGACCCTCTCGAGCCTCACCAGTGACATTGGTGACACGAAGCACAGCCTGTGCACTAAGAGGTGAGAGG
           Consensus ttcctttttctgaagtttttcccaccaagctaaaacatcattttctgaagttacaatatt...c....g.cgtaa...g.g.tcgtgtcatgaacccgtaacaagaccctctcgagcctcaccagtgacattggtgacacgaagcacagcctgtgcactaagag........
```

Fig. S12 Optimization of genomic DNA template input for ddPCR. A – Each column represents a single well of ~18,000 droplets generated from 20-µl reaction mix containing indicated amount of Col-0 genomic DNA and a single set of primers, targeting *DCL1* gene. **B** – The linearity of the dynamic range of ddPCR assays presented in (A).

A

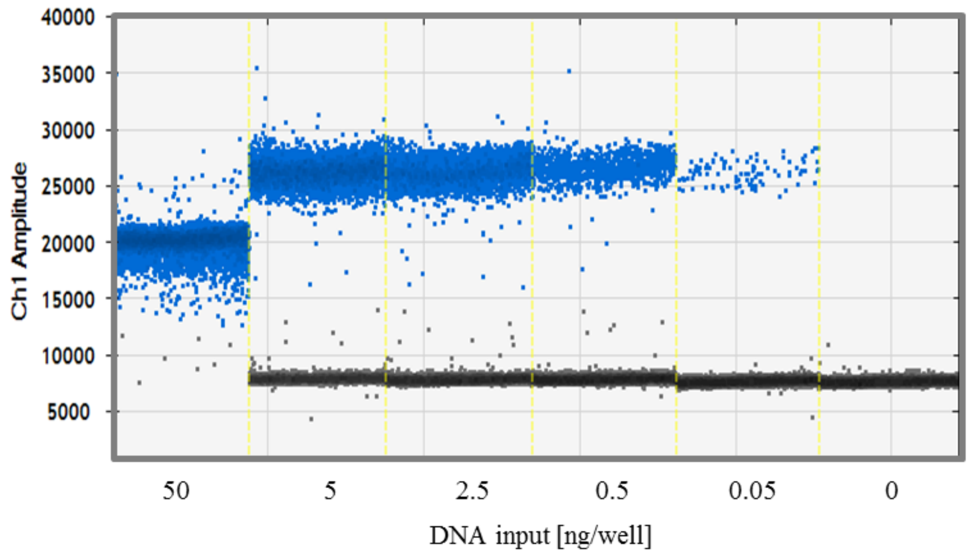

B

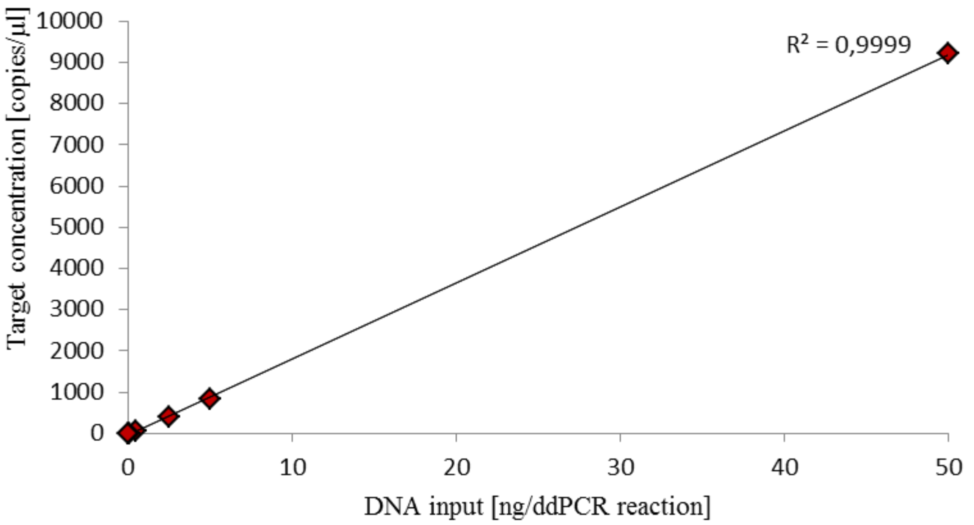

Fig. S13 Optimization of primer annealing temperatures for ddPCR. A – Separation of positive and negative droplets at a range of primer annealing temperatures (56-60 °C). Each column (separated by yellow lines) represents a single well of ~18,000 droplets generated from 20-µl reaction mix containing 1 ng of Col-0 genomic DNA and a single set of primers, targeting *DCL1, HDA15, MSH2, AT3G18530, AT3G18535 or BRC1* gene (as indicated on the bottom of panel B). **B** - The target concentrations detected in the assays presented in (A). The error bars indicate the Poisson 95% confidence intervals.

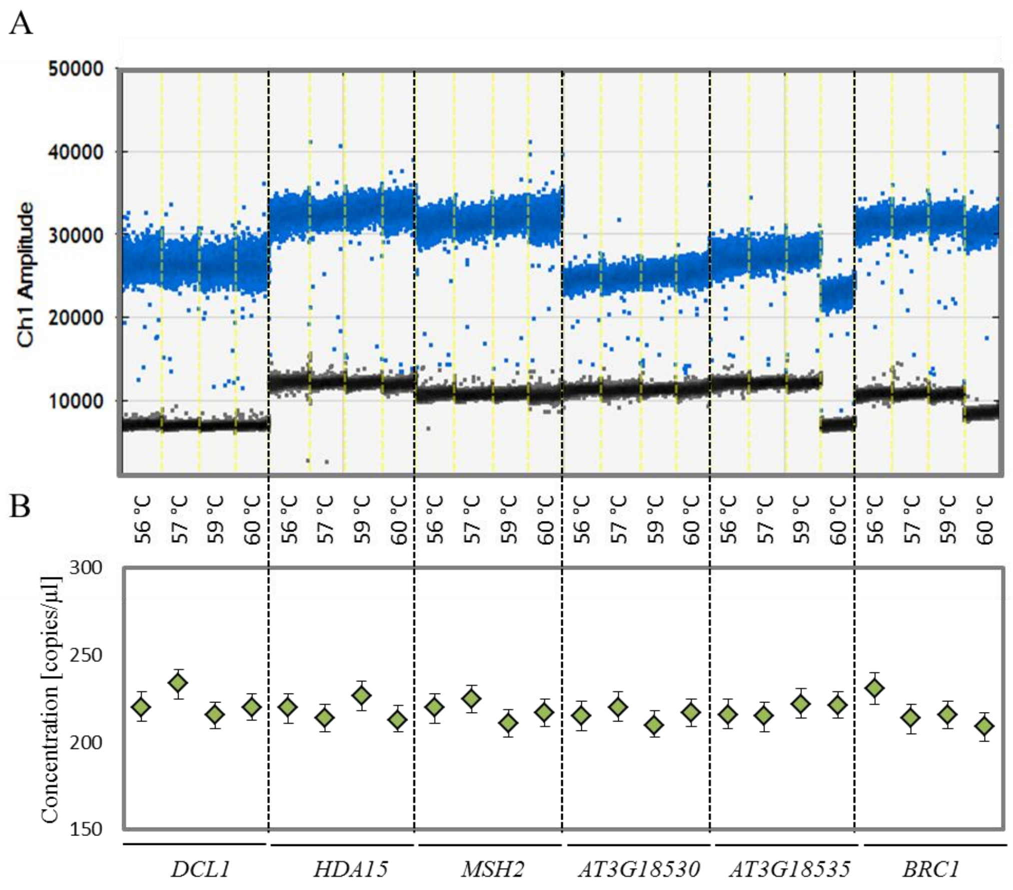

Table S2. Sequences of MLPA probes. Each probe consists of two half probes design to hybridize to adjacent positions of the target genomic DNA. Each half probe consists of a target-specific sequence (red), universal primer sequence (blue) and (optional) stuffer sequence (black).

PROBE	GENE	TARGET-SPECIFIC REGION	LEFT HALF PROBE	RIGHT HALF PROBE
mlpaA	*HDA15*	chr3:6363635-6363683	GGGTTCCCTAAGGGTTGGAcgctactactaGCCTTCACAATAATGCAGCCGTTG	CTGCATTAGTAGCACAAGCAGCAGGatctacTCTAGATTGGATCTTGCTGGCGC
mlpaB	*MSH2*	chr3:6368803-6368850	GGGTTCCCTAAGGGTTGGAcgctactactattagtagaattgatgccacctttCAGTGTCAATGTGAGCGCTGACA	TGGAAGTTTGCCACACCAACAGTGTatgtatctaatggtcaaactaaatctacTCTAGATTGGATCTTGCTGGCGC
mlpaC		chr3:6370464-6370519	GGGTTCCCTAAGGGTTGGAcgctactactattagtaCTAGCTGGTCAAGATCTACAGAGCACTC	AACCAAATCGATGAACTTTCCAAGGTGAaaactaaatctacTCTAGATTGGATCTTGCTGGCGC
mlpaD	*AT3G18530*	chr3:6373869-6373919	GGGTTCCCTAAGGGTTGGAcgctactactattagtagaattgatgccacctttCAGCCAAGCAGTTGGTTTTGATGATAC	ACATGACAACCATGAAGAAACAGAGGCTaatgtatctaatggtcaaactaaatctacTCTAGATTGGATCTTGCTGGCGC
mlpaE		chr3:6375192-6375245	GGGTTCCCTAAGGGTTGGAcgctactactattaGCATTGGCAAAATTGGTGTATCTGC	GATTACAAGGCGTTATATGCCTGAAAAGCaatctacTCTAGATTGGATCTTGCTGGCGC
mlpaF	*AT3G18535*	chr3:6375946-6376000	GGGTTCCCTAAGGGTTGGAcgctactactattagtagaattgatgccacctttCAGCCAAGCAGTTGGTTTTGATGATAC	ACATGACAACCATGAAGAAACAGAGGCTaatgtatctaatggtcaaactaaatctacTCTAGATTGGATCTTGCTGGCGC
mlpaG		chr3:6376454-6376505	GGGTTCCCTAAGGGTTGGAcgctaTAACTTGTGCAAGGTGCCAACGTTG	CGGGTGTCAAACCATAGAACAAAGATGTCTAGATTGGATCTTGCTGGCGC
mlpaH	*BRC1*	chr3:6384247-6384298	GGGTTCCCTAAGGGTTGGAcgctactactattagtagaattGGACACGAGATCGTAGGATGAGACTCT	CGCTAGATGTCGCCAAAGAGTTGTTaatggtcaaactaaatctacTCTAGATTGGATCTTGCTGGCGC
ctrl1	*DCL1*	chr1:23416-31120	GGGTTCCCTAAGGGTTGGAcCAAGAATCATACCTCAGTGAGGTGGTGT	CACTCTTGCAATGTGAGCTTCTGGATCTAGATTGGATCTTGCTGGCGC
ctrl2	*AT4G21580*	chr4:11475719-11477768	GGGTTCCCTAAGGGTTGGAcgctactactattCTTTCCCTGAAGTGGCATGCACT	GTTTGGTCTACTGTCTTCATGATGGGCatctacTCTAGATTGGATCTTGCTGGCGC
ctrl3	*APG10*	chr2:15193694-15195538	GGGTTCCCTAAGGGTTGGAcgctactactattagtagaCTTTCCTTCAACGTCTACGCCATG	CACCAGAAACTCATCTGAAAATCCTCCaactaaatctacTCTAGATTGGATCTTGCTGGCGC
ctrl4	*PFD5*	chr5:7847361-7847414	GGGTTCCCTAAGGGTTGGAcgctactactattagtagaattgatgccTGGCAGATGAAGCTGGAATGGTCTT	GCAGGCCAAAGTTAAACAGTTAACAGCTGaatggtcaaactaaatctacTCTAGATTGGATCTTGCTGGCGC
ctrl5	*PS2*	chr1:27465468-27465522	GGGTTCCCTAAGGGTTGGAcgctactactattagtagaattgatgccaccttttcagcGGTTCGAGTTGATCTCAGAGAAGAGTTC	GCTAATTCCGAGATGTTCAACGATGGTtttgcgaaatgtatctaatggtcaaactaaatctacTCTAGATTGGATCTTGCTGGCGC

Table S3. Gene specific primers used for ddPCR assays.

GENE	primer	sequence
HDA15	dd01	5’- GTGCTGATTGTGGACTGGGTA-3’
	dd02	5’-GGACATCCTGCAACGGTAGA-3’
MSH2	dd03	5’-GACACTCCACGCAGCTAACA -3’
	dd04	5’- ATCTTTGCCCGTGTAGGAGC-3’
AT3G18530	dd05	5’-TGTCTGTGACTGCACGAGGA-3’
	dd06	5’-GTGGGGGAGTTTGTGTCTCA-3’
AT3G18535	dd07	5’-CGACGGCGTTCTCGTTGTTT-3’
	dd08	5’- TGGATGTCCCTTTCTGGTAGC-3’
BRC1	dd09	5’- CACTGAGCCCTCGGAAACTA -3’
	dd10	5’- ACGATCTCGTGTCCCTTTGG-3’
DCL1	dd11	5’- TCTCACGCAGCGTTCCTAAG-3’
	dd12	5’- TGTACCAGGCACAGCATCAA-3’
